# Adapting global evidence-based practice guidelines to the Egyptian healthcare context: the Egyptian Pediatric Clinical Practice Guidelines Committee (EPG) initiative

**DOI:** 10.1186/s42269-023-01059-0

**Published:** 2023-06-13

**Authors:** Ashraf Abdel Baky, Tarek E. I. Omar, Yasser Sami Amer, the Egyptian Pediatric Clinical Practice Guidelines Committee (EPG)

**Affiliations:** 1 Pediatrics Department, Faculty of Medicine, Ain Shams University, Cairo, Egypt; 2 Pediatrics Department, MTI University, Cairo, Egypt; 3 Pediatrics Department, Armed Forces College of Medicine (AFCM), Cairo, Egypt; 4 Egyptian Pediatric Clinical Practice Guidelines Committee (EPG), Cairo, Egypt; 5 Pediatrics Department, Faculty of Medicine, Alexandria University, Alexandria, Egypt; 6 Pediatrics Department, Quality Management Department, King Saud University Medical City, Riyadh, Saudi Arabia; 7 Research Chair for Evidence-Based Health Care and Knowledge Translation, Family and Community Medicine Department, College of Medicine, King Saud University, Riyadh, Saudi Arabia; 8 Adaptation Working Group, Guidelines International Network, Perth, Scotland

**Keywords:** Practice guidelines, Guideline adaptation, Guideline methodology, Guideline program, Adapted-ADAPTE, Pediatrics, Evidence-based pediatrics, Evidence-based medicine, Knowledge translation, National Guidelines, Egypt

## Abstract

**Background:**

In Egypt, academic organizations, professional societies, and research groups develop clinical practice guidelines (CPGs) in order to improve patient quality care and safety. Although important improvements have been made over the past years, many of these consensus-based guideline documents still lack the transparency and methodological rigor of international standards and methodologies recommended by reference evidence-based healthcare and guideline organizations like the Guidelines International Network.

**Main body of the abstract:**

In the Egyptian Pediatric Clinical Practice Guidelines Committee (EPG), we have adopted one of the CPG formal adaptation methodological frameworks named the ‘Adapted ADAPTE’, relevant CPG resources (e.g., the Appraisal of Guidelines for Research and Evaluation or AGREE II Instrument), and involved key stakeholders including clinical and healthcare topic experts and guideline methodologists in producing 32 trustworthy national evidence-based CPGs and one protocol customized to the healthcare context and services provided for Egyptian children. An EPG online website was launched to make these CPGs available and accessible as CPG summaries for pediatricians and relevant healthcare providers.

**Short conclusion:**

The lessons learned, enablers, challenges, and solutions relevant to Egyptian National Pediatric CPGs identified in this paper could be used to address and enrich the debate on pediatric high-quality CPGs, especially for countries of similar contexts and systems.

**Supplementary Information:**

The online version contains supplementary material available at 10.1186/s42269-023-01059-0.

## Background

Clinical practice guidelines (CPGs) are statements that include recommendations for improving patient care. A systematic review of evidence and an assessment of the benefits and costs of alternative care options inform these statements (Institute of Medicine (US) Committee on Standards for Developing Trustworthy Clinical Practice Guidelines 2011).

High-quality evidence-based CPGs are known to support the clinical decisions of relevant healthcare providers and to improve patient outcomes (Djulbegovic et al. 2019; Liu et al. 2021).

Evidence-based methodologies for CPG production include mainly de-novo development (when there are no published CPGs for the target health topic) and adaptation (when there are existing and published one or more eligible CPGs) (Dizon et al. 2016).

Adaptation of CPGs was defined by the Guidelines International Network (GIN) (and the former ADAPTE Collaboration) as “the systematic approach to the modification of a guideline(s) or recommendation(s) produced in one cultural and organizational setting for application in a different context. Adaptation may be used as an alternative to de novo guideline development (e.g., for customizing (an) existing guideline/s to suit the local context)” (Fervers et al. 2011; The ADAPTE Collaboration 2009).

Several methodological frameworks for CPG adaptation were published based on the original ADAPTE Process and/or based on other published CPG methodologies [e.g., the Grading of Recommendations Assessment, Development, and Evaluation or (short GRADE)], CPG tools [e.g., the Appraisal of Guidelines for Research and Evaluation II Instrument (short AGREE II)], or CPG standards [e.g., NAM (former IOM) Standards and GIN Standards]. Yao et al. (2022) reported that 12 CPG adaptation approaches were based on the ADAPTE.

Wang et al. (2018) coined the term ‘formal adaptation’ of CPGs and described it as formal when it is conducted using a CPG adaptation group and an established methodological framework.

To date, there have been several institutional and national CPG adaptation initiatives in the Eastern Mediterranean Region. One of the early CPG formal adaptation methodological frameworks that were applied in the Arab Republic of Egypt was the ADAPTE and one of its modified versions: The ‘Adapted ADAPTE’ (Amer et al. 2015; Alshehri et al. 2023).

The ADAPTE process included three phases (i.e., setup, adaptation, and finalization), 9 modules, and 24 steps. The ‘Adapted ADAPTE’ was proposed by the founding members of the Alexandria Center for Evidence-Based Clinical Practice Guidelines at Alexandria University, Egypt and it included the same overall framework of the original ADAPTE in addition to 3 modified tools, 3 new tools, and 4 alternative steps (Amer et al. 2015).

In June 2018, The Egyptian Pediatric Clinical Practice Guidelines Committee (short EPG) was established and conducted several strategic planning meetings and discussions and launched the first pediatric national CPG program with the Supreme Council of Egyptian University Hospitals (Committee and [Internet]. 2022).

### Aim of the study

The aim of this study is to report and share the experience, enablers, and barriers of the EPG national CPG adaptation program.

## Main text

### The EPG foundation, organization, and methodology

The Egyptian Pediatric CPGs Committee (EPG) marks the first national and collaborative initiative for the generation of Pediatric CPGs using an evidence-based methodology in Egypt.

After the foundation of the EPG under the auspices of the Supreme Council of Egyptian University Hospitals and the Armed Forces College of Medicine, it added 5 EPG subcommittees for (i) strategic planning, (ii) CPG advisory (methodology), (iii) implementation, (iv) publication and research, and (v) quality control.

The strategic plan for national pediatric evidence-based CPGs included: (i) identifying the national healthcare priorities (high-priority health topics for CPGs) in the field of pediatrics and child health; (ii) identifying a scientifically rigorous and evidence-based methodology for CPGs; and (iii) scope of function, roles, and responsibilities of the members of the EPG and the subspecialty CPG groups.

**Fig. 1:**
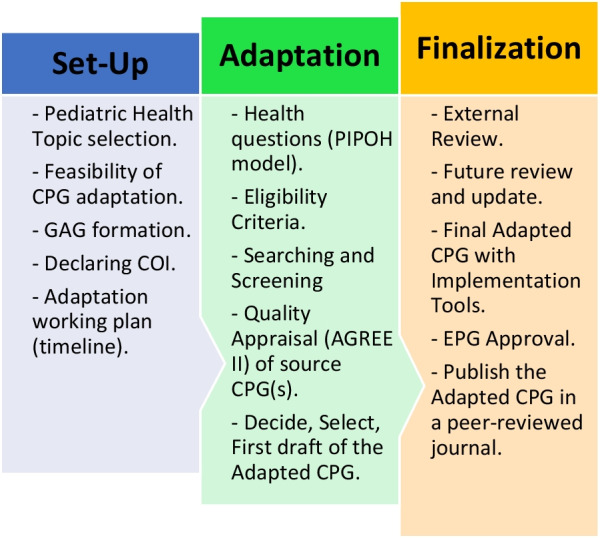
A summary of the Adapted ADAPTE CPG adaptation process. The ‘Adapted ADAPTE’ process for CPG adaptation is divided into the setup phase, the adaptation phase, and the finalization phase. *AGREE II* The Appraisal of Guidelines for Research and Evaluation II Instrument, *CPGs* clinical practice guidelines, *COI* conflict of Interests, *EPG* Egyptian Pediatric Clinical Practice Guidelines Committee, *GAG* Guideline Adaptation Group

The EPG decided to use the CPG adaptation methodology, specifically the ‘Adapted ADAPTE’ methodological framework. The Adapted ADAPTE CPG adaptation process is summarized in Fig. 1.

The founding members reached out and recruited representative faculty staff from the Departments of Pediatrics of 14 Egyptian Universities and one National Research Centre, including, in alphabetical order, the faculties of medicine at the universities of Ain Shams, Al-Azhar, Alexandria, Assiut, Benha, Cairo, Helwan, Mansoura, Menoufia, Minia, October 6, Port Said, Suez Canal, and Zagazig, as well as the Ministry of Health (Additional file 1).

Later on with the progression of the initiative all Egyptian universities participated and contributed to different phases of the CPG adaptation projects.

A representative working group was established for each Pediatric subspecialty (e.g., Pediatric Endocrinology, Gastroenterology, Hematology, Neonatology, Nephrology, Neurology, Respiratory, Allergy, and Immunology, etc.) with a clinical lead and several contributors from multiple universities in the same subspecialty (El-Mazary 2022).

Each EPG working group was encouraged to work on one CPG adaptation project annually for one of the pediatric national health priorities as relevant to the current Pediatric and Child Healthcare Services available and applicable to the actual Egyptian Healthcare context.

The CPG adaptation project involved the collaboration of two main distinct and independent groups: (1) The *Guideline Adaptation Group (or GAG)* (in contrast to the known term Guideline Development Group or GDG that is saved for groups that use the de-novo development methodology), and (2) *The External Review Group (or ERG).*

Each GAG was divided into two subgroups: (i) The Clinical expert subgroup, which includes the Clinical lead of the GAG and its members nominated by the related EPG Pediatric subspecialty group with the EPG Chairman, and (ii) The CPG Methodology supervision subgroup that includes faculty staff and pediatricians with expertise as CPG Methodologists and working experience with the Adapted ADAPTE.

Most members of the EPG CPGs’ GAGs and ERGs are staff of Egyptian Universities, University Hospitals, National Research Centres, the Ministry of Health, or their clinical and methodological networks. They have contributed to the EPG CPG projects on a voluntary basis not related to or funded by any pharmaceutical company, industrial body, or research grants. Some members of the ERGs were international clinical or methodological experts. The declaration of the conflict of interests of all contributors was transparent.

The recruitment process for EPG members was open to any interested child healthcare provider primarily pediatricians and others like clinical pharmacists, and nurses as relevant to the specific health topics representing university hospitals and different healthcare sectors and facilities in Egypt.

### The EPG deliverables: Evidence-Based Pediatric Practice Guidelines for Egypt

The EPG has completed five waves of parallel CPG adaptation projects from 2018 to date that has produced 32 adapted national Evidence-Based CPGs for high-priority pediatric health topics in Egypt, in addition to a consensus-based national protocol for COVID-19 (including four versions so far) (Table 1).

**Table 1:** List of pediatric health topics included in the EPG National Guideline Adaptation Program (Korraa et al. 2022; Abdel Baky et al. 2022; Moustafa et al. 2020, 2021, 2023a, b)

CPG topic	EPG subspecialty groups	Guideline registration number	Adaptation phase	Publication status (DOI if published)	CPG summary uploaded^a^
*First wave*					
1. Asthma	Pulmonology	PREPARE-2023CN114	Approved	*In progress*	Yes
2. Neonatal Jaundice	Neonatology	PREPARE-2023CN217	Approved	https://doi.org/10.21608/anj.2022.121921.1055	Yes
3. Diabetic Ketoacidosis (DKA)	Endocrinology	PREPARE-2023CN016	Approved	*In progress*	No
4. Urinary Tract Infection (UTI)	Nephrology	PREPARE-2023CN218	Approved	https://doi.org/10.1186/s43054-021-00073-z	Yes
5. Complementary Feeding	Clinical Nutrition	PREPARE-2023CN219	Approved	*In progress*	No
*Second wave*					
6. Acute Childhood Seizures	Neurology	PREPARE-2023CN220	Approved	*In progress*	Yes
7. Acute Gastroenteritis	Gastroenterology	PREPARE-2023CN221	Approved	*In progress*	No
8. Bronchiolitis	Pulmonology	PREPARE-2023CN222	Approved	https://doi.org/10.1186/s43054-021-00094-8	Yes
9. Cow Milk Protein Allergy	Clinical Nutrition	PREPARE-2023CN223	Approved	*In progress*	No
	Gastroenterology				
	Allergy, Immunology, and Rheumatology				
10. Iron Deficiency Anemia	Clinical Nutrition	PREPARE-2023CN224	Approved	*In progress*	Yes
	Hematology				
11. Screening and Prevention of Type 2 Diabetes	Endocrinology	PREPARE-2022CN813	Adaptation	*Pending till approval*	No
12. Diagnosis and Treatment of Type 2 Diabetes	Endocrinology	PREPARE-2023CN271	Adaptation	*Pending till approval*	No
*Third wave*					
13. Community-Acquired Pneumonia	Pulmonology	PREPARE-2023CN225	Approved	*In progress*	No
14. Acute Hemolytic Crisis	Hematology	PREPARE-2022CN805	Approved	*In progress*	Yes
15. Non-traumatic acute altered level of consciousness	Neurology	PREPARE-2023CN234	Approved	*In progress*	No
16. Shock	Critical Care	PREPARE-2023CN236	Approved	*In progress*	No
17. Early Onset Neonatal Sepsis	Neonatology	PREPARE-2023CN226	Approved	*In progress*	No
*Fourth wave*					
18. Familial Mediterranean Fever (FMF)	Allergy, Immunology, and Rheumatology	IPGRP-2022CN157	Approved	*In progress*	Yes
19. Patent Ductus Arteriosus (PDA)	Cardiology	PREPARE-2023CN227	Adaptation	*Pending till approval*	No
20. Intravenous Fluid Therapy	Gastroenterology	PREPARE-2023CN229	Approved	*In progress*	No
21. Helicobacter Pylori Infection	Gastroenterology	IPGRP-2022CN137	Approved	*In progress*	Yes
22. Bleeding in Isolated Thrombocytopenia	Hematology	PREPARE-2022CN791	Approved	*In progress*	Yes
23. Enteral Feeding for Low Birth-Weight Infants	Neonatology	PREPARE-2023CN230	Approved	*In progress*	Yes
24. Obesity	Clinical Nutrition	PREPARE-2023CN231	Approved	*In progress*	No
	Endocrinology				
25. Chronic Cough	Pulmonology	PREPARE-2023CN249	Finalization	*Pending till approval*	No
*Fifth wave*					
26. Nephrotic Syndrome	Nephrology	IPGRP-2021CN374	Approved	https://doi.org/10.1186/s43054-022-00119-w	No
				https://doi.org/10.1186/s43054-022-00118-x	
27. Systemic Onset Juvenile Idiopathic Arthritis (sJIA)	Allergy, Immunology and Rheumatology	PREPARE-2022CN711	Finalization	*In progress*	No
28. Hypoxic-Ischemic Encephalopathy (HIE)	Neonatology	PREPARE-2023CN235	Finalization	*In progress*	No
29. Allergic Rhinitis	Pulmonology	PREPARE-2022CN671	Finalization	*In progress*	No
	Allergy, Immunology, and Rheumatology				
30. Functional Constipation	Gastroenterology	IPGRP-2022CN156	Finalization	*In progress*	No
31. Blood Transfusion	Hematology	PREPARE-2022CN444	Finalization	*In progress*	No
32. Faltering Growth	Clinical Nutrition	PREPARE-2023CN233	Finalization	*In progress*	No
	Endocrinology				
33. Diabetic Ketoacidosis (DKA) (Update)	Endocrinology	PREPARE-2023CN016	Adaptation	*In progress*	
34. COVID-19 (Protocol)	The first version was by Pulmonology then all EPG groups participated in three successive updated versions	Not applicable	Approved Protocol	https://doi.org/10.1186/s43054-020-00037-9	Yes

^a^Uploaded to the EPG website: http://epg.edu.eg/

### Disseminating and implementing the EPG CPGs

The EPG chairman and members have contributed to the dissemination and implementation of these National CPGs that resulted from the first four waves and the COVID-19 Protocol by conducting several face-to-face training and education oral presentations of the clinical recommendations and implementation tools of the EPG CPGs in local, national, and international conferences throughout the different governorates of Egypt including Alexandria, Assiut, Beheira, Cairo, Damietta, Ismailia, Kafr El Sheikh, Minia, Port Said, Sharqia, Suez, and others (Abdel 2021; Mamdouh 2021; Sayed 2021).

After the COVID-19 pandemic and lockdown were announced, the EPG shifted these activities to online live and recorded webinars and sessions using the official YouTube channel of the EPG (@guidelinescommittee4961) and other social media networks.

Furthermore, as faculty teaching staff, chairpersons of pediatrics or child healthcare departments, senior consultants, or directors in their institutions, EPG members represent clinical and quality champions supporting CPG implementation. Detailed post-implementation findings for each CPG health topic will be reported individually.

The EPG has future plans to evaluate the success of uptaking and implementing its CPGs in real pediatric practice and evaluate their accessibility to the relevant healthcare providers and target users through interventions like (i) Knowledge, Attitude, and Practices (KAP) surveys, (ii) audit and feedback, and (iii) Plan-Do-Check-Act (PDCA) Quality Improvement Cycles.

### Facilitators and barriers to the EPG national initiative

#### Facilitators

Despite the fact that the COVID-19 pandemic posed challenges to education, research, and other projects and services, the EPG different GAG clinical and methodology members expressed great enthusiasm and resilience by shifting all of the face-to-face meetings and activities to online Zoom meetings and a set of WhatsApp groups for each GAG in addition to the main EPG group (Lieneck et al. 2021; Doyumgaç et al. 2021; Zhang et al. 2022).

Members of the EPG and its working groups are faculty staff with expertise and skills in teaching, training, and medical education in different scenarios and settings. They are practicing physicians and affiliated with different university hospitals and clinics cross-cutting throughout the country (Bank et al. 2019; Mortagy et al. 2022; Bannister et al. 2010; Bassiouny and Elhadidy 2022; Abdel Baky et al. 2021).

The diversity of generations within the CPG groups provided an invaluable opportunity for the exchange of ideas and experiences that was characterized by the transfer of clinical expertise and lessons learned from the senior to the junior in addition to supporting the junior to the senior in information technology, internet, and data management skills (O’Doherty et al. 2019; Bridges et al. 2011).

The EPG has an executive core group that oversees, organizes, and follows up on the initiation, timeline, and sustainability of different activities like identifying high-priority health topics in each pediatric subspecialty, registration of CPGs, phases of each CPG project, submission of the first draft of the CPG full documents, presentation, and discussion meetings between GAGs and ERGs.

Different EPG GAGs invited relevant healthcare specialties to participate in CPG projects, as feasible, like clinical pharmacists and nurses.

The General Authority for Healthcare Accreditation & Regulation (GAHAR) was recently established in 2018 as part of the Universal Health Insurance System in Egypt. GAHAR published seven handbooks of accreditation standards covering different healthcare services throughout which it recommended adherence to CPGs, protocols, and policies and procedures (Mansour et al. 2021, 2020, 2023).

#### Barriers

##### Resource barriers

Clinical practice guidelines are extremely hard to implement in daily practice due to a lack of resources for patients particularly the affordability and accessibility of high-cost medicines (e.g., chemotherapy, biologic therapy, and others).

Lack of financial support dedicated to developing, disseminating, and implementing National CPGs to cover costs like design and printing and article processing fees for CPGs and other potential implementation resources such as National CPGs’ website or database maintenance and CPG mobile Apps.

##### System barriers

There is lack of a national agreement on the process of development, adaptation, official approval, Implementation, revision and update, and auditing of National Guidelines in Egypt.

There is no national body or center assigned to develop, revise, evaluate, validate, and/or officially approve National Guidelines in Egypt similar to the examples of the National Evidence-Based Medicine Center of the Saudi Health Council in Saudi Arabia, the Ministry of Public Health in Qatar, the National Authority for Assessment and Accreditation in Healthcare in Tunisia, the National Institute for Health and Care Excellence (NICE) in the United Kingdom, and the National Health and Medical Research Council (NHMRC) in Australia (Alshehri et al. 2023).

The health system in Egypt is fragmented into multiple healthcare service providers including the public and private sectors. The public sector has mainly the Ministry of Health and Population (MOHP), Curative Care Organization (CCO), Teaching hospitals and institutes organization (THIO), Health Insurance Organization (HIO), Ministry of Higher Education (MOHE) (University Hospitals), and Ministry of Defense (MoD) and Ministry of Interior (MoI). The private sector has the Private medical insurance, Household out-of-pocket payments, and Non-Governmental Organizations. This pluralistic healthcare system poses great challenges to implementing National Guidelines in Egypt. Universal health insurance (UHI) is a new entity established to provide Universal Health Insurance services for all Egyptians and is expected to address some of these barriers (Saleh 2006; Wanis 2015; Kanavos et al. 2020; Fasseeh et al. 2022).

There is lack of integration and collaboration with relevant national initiatives like the Health Technology Assessment (HTA) Institutionalization by The Egyptian Authority for Unified Procurement, Medical Supply and the Management of Medical Technology (UPA) in collaboration with NICE International (Pinilla-Dominguez et al. 2022; Glasziou et al. 2011).

##### Research barriers

Despite the two collaborative Egyptian registries that were established in 2014, The National Registry for Egyptian Pediatric Neuro-muscular Diseases (EGYPT PED-NMD) and the National Egyptian Network Pediatric Stroke and Hemiplegia Registry (NENPSHR), there is a lack of relevant national registries for other high-priority pediatric health topics that in turn disable our estimates for diagnostic and management difficulties in these topics (Hassanein and El-Sobky 2022).

We have no sufficient multicenter studies from which reasonable conclusions could be derived and included in the recommendations of Egyptian CPGs. The WHO/EMRO has called upon the Governments in the Eastern Mediterranean Region and international funding agencies to increase supporting health research and scientific production (Mandil et al. 2018).

Moreover, there is no financial funding from any source for our EPG National Guidelines Initiative till now which of course limits our ability to research, develop evidence-based recommendations, and do effective implementation planning and execution.

Some of the relevant international source CPGs did not grant our EPG working groups permission for adaptation or did not respond. Others have asked for payments for the permission for adaptation of their CPGs which was unaffordable.

##### Attitudinal barriers

The role of the expert opinion is still dominating over evidence-based healthcare by healthcare professionals and providers. This is often reflected on the disagreement of senior physicians (e.g., pediatricians) with the CPG evidence-based recommendations as they fear that CPGs adherence may limit their clinical autonomy, flexibility and individualized approach (Radwan and Adawy 2019; Abdel-Kareem et al. 2019; Shehata et al. 2015).

##### Patient barriers

Patients do not want to conform to treatment guidelines as they are often concerned with the high-cost, side effects, and/or scarcity of medications.

##### Strengths and limitations

This review does not report the different tools and templates included in the Adapted ADAPTE methodological framework since these were reported in the key article of this methodology and in published ‘Adapted ADAPTE’ EPG CPGs (Amer et al. 2015; Korraa et al. 2022; Abdel Baky et al. 2022; Moustafa et al. 2020, 2021, 2023a, b).

This is the first reported national initiative for high-quality pediatric CPGs in Egypt using a formal CPG adaptation methodological framework that presented the enablers, challenges, and potential solutions.

#### Recommendations, solutions, and the way forward

We are, on behalf of the EPG, proposing the following recommendations and solutions to address the aforementioned barriers to National Pediatric CPGs in Egypt and to open them for discussion in countries of similar contexts and systems:

- Establish an independent National Evidence-Based Healthcare body or center dedicated to sustaining the de-novo development, adaptation, implementation, auditing, and revision and update of National Guidelines and Protocols with the appropriate authority and funding.
- Apply and sustain a membership of this proposed national center in relevant international organizations [e.g., Guidelines International Network (GIN) and the International Network of Agencies for Health Technology Assessment (INAHTA)].
- Collaborate with similar national guidelines centers in the Eastern Mediterranean Region to build capacity and exchange experiences with consideration of the similarities and differences between the health systems.
- Integrate the EPG CPGs into the GAHAR Accreditation Standards that are related to child healthcare services and facilities.
- Integrate formal training, modules, and courses of evidence-based healthcare and CPG tools and skills into the curricula of undergraduate and postgraduate students of Health Colleges and Faculties like Medicine, Pharmacy, Nursing, etc. [a good example is the recently launched International Guideline Development Credentialing & Certification Program (or INGUIDE)] (INGUIDE 2023).
- Integrate and align relevant national initiatives to achieve the balance between clinical evidence through CPGs and economic evidence through HTAs (Mason et al. 1999; Lord et al. 2013).

## Conclusions

The lessons learned enablers, challenges, and solutions relevant to Egyptian National Pediatric CPGs identified in this paper could be used to address and enrich the debate on pediatric high-quality CPGs, especially for countries of similar contexts and systems.

## Supplementary Information

**Additional file 1.** Recognition and Honor List.

## Data Availability

Not applicable.

## Abbreviations

AGREE II: The Appraisal of Guidelines for Research and Evaluation II Instrument
COI: Conflicts of Interest
CPG: Clinical Practice Guideline
EBM: Evidence-Based Medicine
EPG: Egyptian Pediatric Clinical Practice Guidelines Committee
ERG: External Review Group
GAG: Guideline Adaptation Group
GAHAR: General Authority for Healthcare Accreditation & Regulation, Egypt
GIN: Guidelines International Network, Perth, Scotland
HTA: Health Technology Assessment
INAHTA: International Network of Agencies for Health Technology Assessment
NHMRC: National Health and Medical Research Council, Australia
NICE: National Institute for Health and Care Excellence, United Kingdom
WHO-EMRO: World Health Organization-Regional Office for the Eastern Mediterranean
