# Supplementary material for: Adapting global evidence-based practice guidelines to the Egyptian healthcare context: the Egyptian Pediatric Clinical Practice Guidelines Committee (EPG) initiative
Source: Bull Natl Res Cent. 2023 Jun 13;47(1):88. doi: 10.1186/s42269-023-01059-0 (PMC10262930; doi:10.1186/s42269-023-01059-0)

Recognition and Honor List

The authors wish to send their highest regards in recognition and acknowledgment to the outstanding and lasting contributions to the Egyptian Pediatric Clinical Practice Guidelines Committee (EPG) Initiative from the following members of the EPG Clinical Practice Guideline Adaptation and External Review Groups representing senior pediatricians and faculty staff of Departments of Pediatrics, Faculties of Medicine of Egyptian Universities, University Hospitals, National Research Centre, Armed Forces College of Medicine, Ministry of Health and Population, Health Insurance Organization in addition to the relevant national and international notable topic experts and contributors.

Contents

Members, Guideline Adaptation Groups/ Pediatric Subspecialty Groups 3
- Allergy, Immunology, and Rheumatology 3
- Cardiology 5
- Clinical Nutrition 6
- Critical Care and Emergency Medicine 7
- Endocrinology 8
- Gastroenterology 10
- Hematology 12
- Neonatology 14
- Nephrology 16
- Neurology 18
  - National 18
  - International 19
- Pulmonology 19
- Additional Contributors *(other than Pediatrics)* 21

Members, Guideline Methodology Supervision Group 22

Members, External Review Groups 22
- Clinical Content Reviewers 22
  - Allergy, Immunology and Rheumatology 22
  - Cardiology 23
  - Critical Care/ Emergency Medicine 23
  - Endocrinology 24
  - Gastroenterology 24
  - Hematology 25
  - Neonatology 26
  - Nephrology 26
  - Neurology 27
  - Pulmonology 27
  - Additional Contributors *(other than Pediatrics)* 28
- Methodology Reviewer 28

EPG Group Photos 29

Members, Guideline Adaptation Groups/ Pediatric Subspecialty Groups

(Each group is arranged in alphabetical order)

Allergy, Immunology, and Rheumatology

- Prof. Ashraf Abdel Baky *(Lead)*

  Department of Pediatrics, Ain Shams University (ASU)

  Department of Pediatrics, Armed Forces College of Medicine (AFCM)

  Department of Pediatrics, Modern University for Technology & Information (MTI) University

- Ass. Prof. Ali Sobh *(Member)*

  Department of Pediatrics, Mansoura University (MANS)

- Dr. Amira Elhatab, Lecturer *(Member)*

  Department of Pediatrics, Ain Shams University (ASU)

- Prof. Ashraf Ahmed Galal *(Member)*

  Department of Pediatrics, Alexandria University (ALEXU)

- Prof. Dalia H. El- Ghoneimy *(Member)*

  Department of Pediatrics, Ain Shams University (ASU)

- Ass. Prof. Eman Fahmy *(Member)*

  Department of Pediatrics, Sohag University

- Dr. Ghada Abdel Haleem Shousha, Lecturer *(Member)*

  Department of Pediatrics, Ain Shams University (ASU)

- Ass. Prof. Ghada El Deriny *(Member)*

  Department of Pediatrics, Alexandria University (ALEXU)

- Prof. Hanan Abd El Lateef *(Member)*

  Department of Pediatrics, Ain Shams University (ASU)

- Ass. Prof. Iman Fahmy *(Member)*

  Department of Pediatrics, Suhag University

- Prof. Islam Elbaroudy *(Member)*

  Department of Pediatrics, Cairo University (CU)

- Prof. Maher Ahmed Abdel Hafez *(Member)*

Department of Pediatrics, Tanta University (TU)

- Prof. Mohamed Almalky *(Member)*

  Department of Pediatrics, Zagazig University (ZU)

- Dr. Naglaa Samy Mohamed Osman *(Member)*

  Department of Pediatrics, Assiut University (AUN)

- Dr. Nehal Refaat, Ass. Lecturer *(Member)*

  Department of Pediatrics, Armed Forces College of Medicine (AFCM)

- Ass. Prof. Nesrine Radwan *(Member)*

  Department of Pediatrics, Ain Shams University (ASU)

- Ass. Prof. Rasha El-Owaidy *(Member)*

  Department of Pediatrics, Ain Shams University (ASU)

- Dr. Sally Gouda *(Member)*

  Department of Pediatrics, Ain Shams University (ASU)

- Prof. Sanaa Abd El Rahman Mahmoud *(Member)*

  Department of Pediatrics, Ain Shams University (ASU)

- Prof. Sheren Esam Maher *(Member)*

  Department of Pediatrics, Minia University (MU)

- Prof. Shereen Medhat Reda *(Member)*

  Department of Pediatrics, Ain Shams University (ASU)

- Dr. Walaa Shoman, Lecturer *(Member)*

  Department of Pediatrics, Alexandria University (ALEXU)

- Ass. Prof. Yomna Mohamed Farag *(Member)*

  Department of Pediatrics, Cairo University (CU)

- Prof. Zeinab Awad El- Sayed *(Member)*

  Department of Pediatrics, Ain Shams University (ASU)

Cardiology

- Prof. Alyaa A. Kotby *(Lead)*

  Department of Pediatrics, Ain Shams University (ASU)
- Prof. Aly Abdelmohsen *(Member)*

  Department of Pediatrics, Alexandria University (ALEXU)
- Prof. Amal Elsisi *(Member)*

  Department of Pediatrics, Cairo University (CU)
- Prof. Baher Matta Hanna *(Member)*

  Department of Pediatrics, Cairo University (CU)

  Department of Pediatrics, Armed Forces College of Medicine (AFCM)
- Prof. Duaa M. Raafat *(Member)*

  Department of Pediatrics, Assiut University (AUN)
- Prof. FatmaAlzahraa Mostafa *(Member)*

  Department of Pediatrics, Cairo University (CU)
- Prof. Hala Agha *(Member)*

  Department of Pediatrics, Cairo University (CU)
- Prof. Hala Elmarsafawy *(Member)*

  Department of Pediatrics, Mansoura University (MANS)
- Prof. Hebatallah Attia *(Member)*

  Department of Cardiology, Ain Shams University (ASU)
- Prof. Magdy Mahmoud *(Member)*

  Department of Pediatrics, Minia University (MU)
- Dr. Mahmoud Nadder, Ass. Lecturer *(Member)*

  Department of Pediatrics, Alexandria University (ALEXU)
- Prof. Maiy El-Sayed *(Member)*

  Department of Cardiology, Ain Shams University (ASU)
- Prof. Mona El Ganzoury *(Member)*

  Department of Pediatrics, Ain Shams University (ASU)
- Dr. Nora Elsamman, Lecturer *(Member)*

  Department of Pediatrics, Ain Shams University (ASU)

- Prof. Sally Ahmed Farid El-Sahrigy *(Member)*

  Pediatrics Department, Medical Research and Clinical Studies Institute, National Research Centre (NRC)
- Prof. Sonia El-Saiedi *(Member)*

  Department of Cardiology, Ain Shams University (ASU)
- Ass. Prof. Yasmin Abdelrazek*(Member)*

  Department of Cardiology, Ain Shams University (ASU)

Clinical Nutrition

- Prof. Sanaa Youssef Shaaban *(Lead)*

  Department of Pediatrics, Ain Shams University (ASU)
- Prof. Afaf Abdel Fattah Tawfik *(Member)*

  National Nutrition Institute
- Dr. Asmaa Sadek A. Sadek *(Member)*

  Department of Pediatrics, Armed Forces College of Medicine (AFCM)
- Dr. Ayah Shabana *(Member)*

  Department of Pediatrics, Armed Forces College of Medicine (AFCM)
- Prof. Ehab Khairy El Khashab *(Member)*

  Department of Pediatrics, Ain Shams University (ASU)
- Prof. Enas Raafat *(Member)*

  Department of Child Health, Medical Research and Clinical Studies Institute, National Research Centre (NRC)
- Prof. Hanna Mohamed Abulghar *(Member)*

  Department of Pediatrics, Cairo University (CU)
- Prof. Mahmoud Rashad *(Member)*

  Department of Pediatrics, Al-Azhar University
- Dr. Manar Mohamed Fathy *(Member)*

  Department of Pediatrics, Zagazig University (ZU)
- Prof. Mourad Alfy Ramzy Tadros *(Member)*

  Department of Pediatrics, Armed Forces College of Medicine (AFCM)

- Dr. Nahed Mohamed Hamdy *(Member)*
  Department of Pediatrics, Armed Forces College of Medicine (AFCM)
- Prof. Osama Mahmoud El-Asheer *(Member)*
  Department of Pediatrics, Assiut University (AUN)
- Dr. Shrouk MoatazAbdallah *(Member)*
  Department of Pediatrics, Cairo University (CU)
- Prof. Somaya Mohamed Abd El-Ghany *(Member)*
  Department of Pediatrics, Al-Azhar University
- Prof. Tayseer Mohamed El Zayat *(Member)*
  Department of Pediatrics, Al-Azhar University
- Ass. Prof. Yasmin Gamal El Gendy *(Member)*
  Department of Pediatrics, Ain Shams University (ASU)

Critical Care and Emergency Medicine

- Prof. Hafez Mahmoud Bazaraa *(Lead)*
  Department of Pediatrics, Cairo University (CU)
- Ass. Prof. Ahmed Rezk Ahmed *(Member)*
  Department of Pediatrics, Ain Shams University (ASU)
- Dr. Alyaa Ahdy AbdEl Aziz *(Member)*
  Department of Pediatrics, Minufiya University
- Prof. Ateyat Abo Elfotoh Ateya *(Member)*
  Department of Pediatrics, Al-Azhar University
- Prof. Azza Ahmed Eltayeb *(Member)*
  Department of Pediatrics, Assiut University (AUN)
- Prof. Dalia A. Abdelrahman *(Member)*
  Department of Pediatrics, Zagazig University (ZU)
- Ass. Professor Effat Hussein Assar *(Member)*
  Department of Pediatrics, Benha University (BU)
- Prof. Hanaa Ibrahim Rady *(Member)*
  Department of Pediatrics, Cairo University (CU)

- Prof. Khaled Talaat Muhammad

  Department of Pediatrics, Tanta University (TU)
- Dr. Marwa Nabil Saad, Ass. Lecturer *(Member)*

  Department of Pediatrics, Cairo University (CU)
- Prof. Mervat Gamal Eldin Mansour *(Member)*

  Department of Pediatrics, Ain Shams University (ASU)
- Prof. Mohamed Mahmood Ahmed Romih *(Member)*

  Department of Pediatrics, Zagazig University (ZU)
- Prof. Nehad Ahmed Karam Abd Elfatah *(Member)*

  Department of Pediatrics, Zagazig University (ZU)
- Dr. Nevin Abdullah Kamel *(Member)*

  Casualty Department, Zagazig University Hospital, Zagazig
- Dr. Noha El-Anwar, Lecturer *(Member)*

  Department of Pediatrics, Cairo University (CU)
- Prof. Sarah Naeem Bartella Hebish *(Member)*

  Pediatric Oncology and Intensive Care Department, National Cancer Institute, Cairo University
- Ass. Professor Shereen Abdel Monem Mohamed *(Member)*

  Department of Pediatrics, Cairo University (CU)
- Ass. Prof. Sondos Mohamed Magdy *(Member)*

  Department of Pediatrics, Ain Shams University (ASU)
- Dr: Tarek Abd El Latef Abd El Aziz, Lecturer *(Member)*

  Department of Pediatrics, Zagazig University (ZU)

Endocrinology

- Prof. Mona Mamdouh Hassan *(Lead)*

  Department of Pediatrics, Cairo University (CU)
- Ass. Prof. Amal Gaber Mohamed *(Member)*

  Department of Pediatrics, Al-Azhar University
- Prof. Amany Kamal El-Hawary *(Member)*

Department of Pediatrics, Mansoura University (MANS)

- Prof. Amina M. Abdel Wahab *(Member)*

  Department of Pediatrics, Suez Canal University (SCU)
- Prof. Ashraf A. Elsharkawy *(Member)*

  Department of Pediatrics, Mansoura University (MANS)
- Prof. Basma Abd-Elmoez

  Department of Pediatrics, Minia University (MU)
- Dr. Eman Elshanawany *(Member)*

  Department of Pediatrics, Benha University (BU)
- Prof. Hanaa Abdellateef Mohamad *(Member)*

  Department of Pediatrics, Assiut University (AUN)
- Dr. Hanan Hassan Aly, Lecturer *(Member)*

  Department of Pediatrics, Ain Shams University (ASU)
- Prof. Hoda Atwa *(Member)*

  Department of Pediatrics, Suez Canal University (SCU)
- Prof. Lubna Fawaz *(Member)*

  Department of Pediatrics, Cairo University (CU)
- Dr. Mariam Nader Moawad, Ass. Lecturer *(Member)*

  Department of Pediatrics, Armed Forces College of Medicine (AFCM)
- Dr. Marian Fares Nashed, Ass. Lecturer *(Member)*

  Department of Pediatrics, Armed Forces College of Medicine (AFCM)
- Ass. Prof. Mona Karem Amin *(Member)*

  Department of Pediatrics, Suez Canal University (SCU)
- Prof. Nora E. Badawi *(Member)*

  Department of Pediatrics, Cairo University (CU)
- Ass. Prof. Nouran Y. Salah El-Din *(Member)*

  Department of Pediatrics, Ain Shams University (ASU)
- Ass. Prof. Omneya Magdy Omar *(Member)*

  Department of Pediatrics, Alexandria University (ALEXU)
- Dr. Ramy Saleh Morsy, Lecturer *(Member)*

Department of Pediatrics, Armed Forces College of Medicine (AFCM)

- Prof. Randa M. Matter *(Member)*

  Department of Pediatrics, Ain Shams University (ASU)
- Ass. Prof. Remon M. Yousef *(Member)*

  Department of Pediatrics, Fayoum University (FU)
- Prof. Sabry M Ghanem *(Member)*

  Department of Pediatrics, Al-Azhar University
- Prof. Safinaz El Habashy *(Member)*

  Department of Pediatrics, Ain Shams University (ASU)
- Ass. Prof. Shaymaa Elsayed Abdel Meguid *(Member)*

  Department of Pediatrics, Alexandria University (ALEXU)
- Prof. Shereen Abdelghaffar *(Member)*

  Department of Pediatrics, Cairo University (CU)
- Prof. Wiam Al Farouk Younis *(Member)*

  Department of Pediatrics, Armed Forces College of Medicine (AFCM)

Gastroenterology

- Prof. Ayman Emil Eskander *(Lead)*

  Department of Pediatrics, Cairo University (CU)
- Prof. Suzan Samir Gad *(Lead)*

  Department of Pediatrics, Suez Canal University (SCU)

  Department of Pediatrics, Suez University (SU)
- Prof. Abeer M. Nour ElDin Abd ElBaky *(Member)*

  Department of Pediatrics, Medical Research and Clinical Studies Institute, National Research Centre (NRC)
- Prof. Ahmed Foad *(Member)*

  Department of Pediatrics, Alexandria University (ALEXU)
- Prof. Ahmed Hamdy *(Member)*

  Department of Pediatrics, Ain Shams University (ASU)
- Dr. Ahmed Hendawy, Lecturer *(Member)*

Department of Pediatrics, Al-Azhar University

- Prof. Ahmed Mohammed Hamdy *(Member)*

  Department of Pediatrics, Ain Shams University (ASU)
- Prof. Amal Mahfouz *(Member)*

  Department of Pediatrics, Alexandria University (ALEXU)
- Dr. Baher Hanaa *(Member)*

  Department of Pediatrics, Cairo University (CU)
- Dr. Ehab W. Abd Elmohsen, Lecturer (*Member)*

  Department of Pediatrics, Armed Forces College of Medicine (AFCM)
- Dr. Eslam Tawfik ElBaroudy (*Member)*

  Department of Pediatrics, Cairo University (CU)
- Prof. Gihan Mohamed Mohamed Bebars *(Member)*

  Department of Pediatrics, Minia University (MU)
- Dr. Hala Hussien Mansour, Lecturer *(Member)*

  Department of Pediatrics, Cairo University (CU)
- Prof. Hanan Fathy *(Member)*

  Department of Pediatrics, Cairo University (CU)
- Dr. Hend Fayez *(Member)*

  Department of Pediatrics, Armed Forces College of Medicine (AFCM)
- Prof. Hosam F. El-Saadany (*Member)*

  Department of Pediatrics, Zagazig University (ZU)
- Prof. Manal Sadek Eldefrawy *(Member)*

  Department of Pediatrics, Benha University (BU)
- Prof. Maha Abou Zekri *(Member)*

  Department of Pediatrics, Cairo University (CU)
- Prof. Marwa Talaat Eldeeb *(Member)*

  Department of Pediatrics, Ain Shams University (ASU)
- Prof. Mohamed Amin *(Member)*

  Department of Pediatrics, Mansoura University (MANS)
- Prof. Mohamed Ezz El Regal *(Member)*

Department of Pediatrics, Mansoura University (MANS)

- Prof. Mohamed Genina *(Member)*

  Department of Pediatrics, Cairo University (CU)
- Prof. Mohammed Nabih Almohammady *(Member)*

  Department of Pediatrics, Cairo University (CU)
- Prof. Mohamed Saad Eldin Eladawy *(Member)*

  Department of Pediatrics, Ain Shams University (ASU)
- Prof. Naglaa Abu Faddan *(Member)*

  Department of Pediatrics, Assiut University (AUN)
- Ass. Prof. Nesrine Radwan *(Member)*

  Department of Pediatrics, Ain Shams University (ASU)
- Dr. Sarah Tarek *(Member)*

  Department of Pediatrics, Cairo University (CU)
- Prof. Shereen Reda *(Member)*

  Department of Pediatrics, Ain Shams University (ASU)
- Dr. Yosra Mohsen Awad, Lecturer *(Member)*

  Department of Pediatrics, Ain Shams University (ASU)

Hematology

- Prof. Galila Mokhtar *(Lead)*

  Department of Pediatrics, Ain Shams University (ASU)
- Prof. Amira Adly *(Member)*

  Department of Pediatrics, Ain Shams University (ASU)
- Prof. Azza Abd El Gawad Tantawy *(Member)*

  Department of Pediatrics, Ain Shams University (ASU)
- Prof. Dina Ezzat *(Member)*

  Department of Pediatrics, Beni-Suef University (BSU)
- Prof. Gehan Lotfy *(Member)*

  Department of Pediatrics, Minia University (MU)
- Prof. Hoda Hassab *(Member)*

Department of Pediatrics, Alexandria University (ALEXU)

- Prof. Ilham Youssry *(Member)*

  Department of Pediatrics, Cairo University (CU)

- Prof. Iman Ragab *(Member)*

  Department of Pediatrics, Ain Shams University (ASU)

- Prof. Laila Sherief *(Member)*

  Department of Pediatrics, Zagazig University (ZU)

- Prof. Marwa Zakaria *(Member)*

  Department of Pediatrics, Zagazig University (ZU)

- Prof. Mervat Abdallah Hesham *(Member)*

  Department of Pediatrics, Zagazig University (ZU)

- Dr. Naglaa Shaheen (Consultant) *(Member)*

  Pediatric Hematology Department, Health Insurance Organization (HIO)

- Ass. Prof. Niveen Salama *(Member)*

  Department of Pediatrics, Cairo University (CU)

- Dr. Nouran Yousef Salah, Lecturer *(Member)*

  Department of Pediatrics, Ain Shams University (ASU)

- Prof. Rasha Abdel-Raouf Afifi *(Member)*

  Department of Pediatrics, Cairo University (CU)

- Prof. Rasha El-Ashry *(Member)*

  Department of Pediatrics, Mansoura University (MANS)

- Ass. Prof. Sara Makkeyah *(Member)*

  Department of Pediatrics, Ain Shams University (ASU)

- Prof. Sherein Abdelhamid Shalaby *(Member)*

  Department of Pediatrics, Helwan University (HU)

- Prof. Sonia Adolf Habib *(Member)*

  Department of Pediatrics, Medical Research and Clinical Studies Institute, National Research Centre (NRC)

Neonatology

- Prof. Abdel-Azeem Mohamed El-Mazary *(Lead)*

  Department of Pediatrics, Minia University (MU)
- Prof. Afaf Abdel Wahab Korraa *(Lead)*

  Department of Pediatrics, Al-Azhar University
- Prof. Iman Fathy Iskander *(Lead)*

  Department of Pediatrics, Cairo University (CU)
- Prof. Mosallam Mohammed Nasser *(Lead)*

  Department of Pediatrics, Al-Azhar University
- Dr. Ahmed Mahmoud Ali Mostafa, Consultant *(Member)*

  Department of Pediatrics, Assiut University (AUN)
- Dr. Ahmed Mahmoud Youssef, Lecturer *(Member)*

  Department of Pediatrics, Armed Forces College of Medicine (AFCM)
- Prof. Ahmed Mohsen AbdElhakeem *(Member)*

  Department of Pediatrics, Al-Azhar University
- Dr. Ayah Mohamed Shabana, Lecturer *(Member)*

  Department of Pediatrics, Ain Shams University (ASU)
- Dr. Dina Essam Rabie, Lecturer *(Member)*

  Department of Pediatrics, Ain Shams University (ASU)
- Ass. Prof. Effat Hussein Assar *(Member)*

  Department of Pediatrics, Benha University (BU)
- Dr. Eman Almorsy *(Member)*

  Department of Pediatrics, Al-Azhar University
- Dr. Esraa Ahmed Elmazzahy, Lecturer *(Member)*

  Department of Pediatrics, Cairo University (CU)
- Prof. Gamal Samy *(Member)*

  Department of Pediatrics, Ain Shams University (ASU)
- Prof. Ghada Ibrahim Gad *(Member)*

  Department of Pediatrics, Ain Shams University (ASU)
- Dr. Hala Fouad *(Member)*

Department of Pediatrics, Misr University for Science & Technology (MUST)

- Prof. Hisham Abd Elsamee Awad *(Member)*

  Department of Pediatrics, Ain Shams University (ASU)
- Dr. Hussein Hanafy *(Member)*

  Department of Pediatrics, Helwan University (HU)
- Ass. Prof. Madiha Abda-Allah Sayed *(Member)*

  Department of Pediatrics, Minia University (MU)
- Ass. Prof. Mohamed Abdel-Kader *(Member)*

  Department of Pediatrics, Misr University for Science & Technology (MUST)
- Prof. Mohamed Mamdouh Gaafar *(Member)*

  Department of Pediatrics, Zagazig University (ZU)
- Dr. Mohammed Abdelshafy *(Member)*

  Department of Pediatrics, Benha University (BU)
- Prof. Mostafa Abdel Azim *(Member)*

  Department of Pediatrics, Benha University (BU)
- Dr. Nefeisa Raafat *(Member)*

  Department of Pediatrics, Assiut University (AUN)
- Dr. Nouran B. AbdAlla *(Member)*

  Department of Pediatrics, Suez Canal University (SCU)
- Prof. Osama Abou El Fotouh El- Fiky *(Member)*

  Department of Pediatrics, Benha University (BU)
- Dr. Rabab Gameel Abdel-Hamed Allam, Lecturer *(Member)*

  Department of Pediatrics, Ain Shams University (ASU)
- Prof. Reem Mahmoud *(Member)*

  Department of Pediatrics, Cairo University (CU)
- Prof. Safaa Shafik Emam *(Member)*

  Department of Pediatrics, Ain Shams University (ASU)
- Ass. Prof. Salah Ibrahim *(Member)*

Department of Pediatrics, Misr University for Science & Technology (MUST)

- Prof. Sameh Tewfik *(Member)*

  Department of Pediatrics, Armed Forces College of Medicine (AFCM)

- Ass. Prof. Walaa Alsharany Abuelhamd *(Member)*

  Department of Pediatrics, Cairo University (CU)

- Dr. Zahraa Ez El-Din *(Member)*

  Department of Pediatrics, Cairo University (CU)

Nephrology

- Prof. Bahia H. Moustafa *(Lead)*

  Department of Pediatrics, Cairo University (CU)

- Ass. Prof. Abeer Selim *(Member)*

  Department of Pediatrics, Medical Research and Clinical Studies Institute, National Research Centre (NRC)

- Prof. Ahmed Badr *(Member)*

  Department of Pediatrics, Cairo University (CU)

- Ass. Prof. Ahmed Hussien *(Member)*

  Department of Pediatrics, Ain Shams University (ASU)

- Prof. Ayman Hammad *(Member)*

  Department of Pediatrics, Mansoura University (MANS)

- Ass. Prof. Dina E. Sallam *(Member)*

  Department of Pediatrics, Ain Shams University (ASU)

- Prof. Doaa Youssef *(Member)*

  Department of Pediatrics, Zagazig University (ZU)

- Dr. Fatma Atia, Lecturer *(Member)*

  Department of Pediatrics, Cairo University (CU)

- Prof. Gamal Taha Soliman *(Member)*

  Department of Pediatrics, Port Said University (PSU)

- Dr. Hend Abdel El Nabi *(Member)*

Department of Pediatrics, Tanta University (TU)

- Prof. Ihab Z. El Hakim *(Member)*

  Department of Pediatrics, Ain Shams University (ASU)
- Dr. Isra El Bahkiry *(Member)*

  Department of Pediatrics, Mansoura University (MANS)
- Prof. Mahmoud M. El-Kersh *(Member)*

  Department of Pediatrics, Alexandria University (ALEXU)
- Dr. Mai Korkor *(Member)*

  Department of Pediatrics, Mansoura University (MANS)
- Dr. Marwa Dagher *(Member)*

  Department of Pediatrics, Mansoura University (MANS)
- Ass. Prof. Marwa Nabhan *(Member)*

  Department of Pediatrics, Cairo University (CU)
- Prof. Moftah Mohamed Rabie *(Member)*

  Department of Pediatrics, Al-Azhar University
- Prof. Mohamed Shouman *(Member)*

  Department of Pediatrics, Medical Research and Clinical Studies Institute, National Research Centre (NRC)
- Dr. Moustafa El Balshy *(Member)*

  Department of Pediatrics, Benha University (BU)
- Dr. Nancy Abdel-Salam *(Member)*

  Department of Pediatrics, Alexandria University (ALEXU)
- Prof. Ragia Marei Ali *(Member)*

  Department of Pediatrics, Ain Shams University (ASU)
- Ass. Prof. Sameh Abdelaziz Ahmed Mansour *(Member)*

  Department of Pediatrics, Al-Azhar University
- Prof. Samuel Makar *(Member)*

  Department of Pediatrics, Cairo University (CU)
- Prof. Sawsan Moselhy *(Member)*

  Department of Pediatrics, Ain Shams University (ASU)

- Prof. Sherein Shalaby *(Member)*

  Department of Pediatrics, Suez Canal University (SCU)

Neurology

National

- Prof. Tarek El Sayed Ismail Omar *(Lead)*

  Department of Pediatrics, Alexandria University (ALEXU)

- Ass. Prof. Abdelsattar Abdallah Elsayeh *(Member)*

  Department of Pediatrics, Al-Azhar University

- Dr. Amr I. Risha, Lecturer *(Member)*

  Department of Pediatrics, Zagazig University (ZU)

- Prof. Ashraf Azmy El-Refaie *(Member)*

  Department of Child Health, Medical Research and Clinical Studies Institute, National Research Centre (NRC)

- Prof. Azza Ahmed Eltayeb

  Department of Pediatrics, Assiut University (AUN)

- Prof. Dalia Abdellatif Adbelrahman *(Member)*

  Department of Pediatrics, Zagazig University (ZU)

- Prof. Ebtesam Hussein El Melegy *(Member)*

  National Institute of Neuromotor System,

  General Organization for Teaching Hospitals and Institutes

- Dr. Fikry Bassiouny, Consultant* *(Member)*

  Ministry of Health and Population (MOHP)

- Prof. Hadeer Mahmoud Gamal Eldin Abdelghaffar *(Member)*

  Department of Pediatrics, Fayoum University (FU)

- Dr. Hammouda Eid El Gazzar, Fellow and Lecturer *(Member)*

  El Beheira Health Directorate, General Organization of Teaching Hospitals, Ministry of Health and Population (MOHP)

- Prof. Hanan Mohamed Ibrahim

  Department of Pediatrics, Ain Shams University (ASU)

- Prof. Hoda Yehia Tomoum *(Member)*

  Department of Pediatrics, Ain Shams University (ASU)

- Prof. Iman Ali Elagouza *(Member)*

  Department of Pediatrics, Ain Shams University (ASU)

- Prof. Lobna Abdel Gawad Mansour *(Member)*

  Department of Pediatrics, Cairo University (CU)

- Ass. Prof. Marwa Abd Elmaksoud *(Member)*

  Department of Pediatrics, Alexandria University (ALEXU)

- Dr. Michael Nabil Halim Tanious, Lecturer *(Member)*

  Department of Pediatrics, Ain Shams University (ASU)

- Prof. Moustafa Zakaria Mohamed *(Member)*

  Department of Pediatrics, Cairo University (CU)

- Prof. Omnia Fathy El Rashidy *(Member)*

  Department of Pediatrics, Ain Shams University (ASU)

- Dr. Raghda M. H. Zaitoun, Lecturer *(Member)*

  Department of Pediatrics, Ain Shams University (ASU)

- Prof. Sahar M. A. Hassanein *(Member)*

  Department of Pediatrics, Ain Shams University (ASU)

International

- Dr. Christopher Rittey, Consultant *(Member)*

  Sheffield Children’s NHS Foundation Trust, UK

Pulmonology

- Prof. Eman Mahmoud Fouda *(Lead)*

  Department of Pediatrics, Ain Shams University (ASU)

- Prof. Mohamed M. Rashad *(Lead)*

  Department of Pediatrics, Benha University (BU)

- Prof. Tarek Hamed *(Lead)*

  Department of Pediatrics, Zagazig University (ZU)

- Prof. Abla Saleh Mostafa *(Member)*

Department of Pediatrics, Cairo University (CU)

- Dr. Ahmad Ata Sobeih, Lecturer *(Member)*

  Department of Pediatrics, Benha University (BU)

- Prof. Ahmed Mohamad Abd Al-Razek *(Member)*

  Department of Pediatrics, Tanta University (TU)

- Dr. Amal Ibrahim Hassanain *(Member)*

  Pediatrics Department, Medical Research and Clinical Studies Institute, National Research Centre (NRC)

- Prof. Dina Hossam Eldine Hamed *(Member)*

  Department of Pediatrics, Cairo University (CU)

- Ass. Prof. Dina Tawfeek Sarhan *(Member)*

  Department of Pediatrics, Zagazig University (ZU)

- Prof. Hala Gouda Elnady *(Member)*

  Department of Child Health, Medical Research and Clinical Studies Institute, National Research Centre (NRC)

- Prof. Hala Hamdi *(Member)*

  Department of Pediatrics, Cairo University (CU)

- Prof. Hoda M. Salah El-Din Metwally *(Member)*

  Department of Pediatrics, Al-Azhar University

- Prof. Magda Hassab Allah Mohamed *(Member)*

  Department of Pediatrics, Al-Azhar University

- Prof. Magda Gobran *(Member)*

  Department of Pediatrics, Al-Azhar University

- Dr. Malak Shaheen *(Member)*

  Pediatrics Department, Ain Shams University (ASU)

- Prof. Mona Mohsen Elattar *(Member)*

  Department of Pediatrics, Cairo University (CU)

- Prof. Mostafa Al-Saeed *(Member)*

  Department of Pediatrics, Assiut University (AUN)

- Prof. Shahenaz Mahmoud Hussein *(Member)*

Department of Pediatrics, Al-Azhar University

- G.M. Prof. Usama Kasam *(Member)*

  Department of Pediatrics, Armed Forces College of Medicine (AFCM)

Additional Contributors *(other than Pediatrics)*

- Prof. Alaa Roushdy *(Member)*

  Department of Diagnostic Radiology, Ain Shams University (ASU)

- Prof. Hayam Refaat Tantawi *(Member)*

  Pediatric Nursing, Faculty of Nursing, Ain Shams University (ASU)

- Prof. Manal Salman *(Member)*

  Department of Pathology, Ain Shams University (ASU)

- Dr. Marwa Sayed Mohammed Moussa, Lecturer *(Member)*

  Department of Diagnostic Radiology, Ain Shams University (ASU)

- Prof. Maysaa Abdallah Saeed *(Member)*

  Tropical Medicine and Infectious Diseases, Zagazig University (ZU)

- Prof. Mohamed-Adel Elgamal *(Member)*

  Department of Congenital and Pediatric Cardiac Surgery, Mansoura University (MANS)

- Dr. Nesreen Mohamad Kamal, Lecturer *(Member)*

  Department of Community Medicine, Cairo University (CU)

- Prof. Noha Mohamed Abdelsalam Sakr *(Member)*

  Department of Community Medicine, Zagazig University (ZU)

- Pharmacist Sara Naeem Bartella Hebish *(Member)*

  Senior Clinical Pharmacist, Pediatric Oncology and ICU Department, National Cancer Institute, Cairo University (CU)

Members, Guideline Methodology Supervision Group

(The authorship group of this review)

- Prof. Ashraf Abdel Baky
- Prof. Tarek E. I. Omar
- Dr. Yasser S. Amer

Members, External Review Groups

Clinical Content Reviewers

Allergy, Immunology and Rheumatology

National

- Ass. Prof. Ghada Al-Deriny

  Department of Pediatrics, Alexandria University (ALEXU)
- Prof. Mahmoud M. El-Zalabany

  Department of Pediatrics, Alexandria University (ALEXU)
- Prof. Mohamed Ezz Al-Regal

  Department of Pediatrics, Mansoura University (MANS)
- Prof. Mostafa El-Hodhod

  Department of Pediatrics, Ain Shams University (ASU)
- Prof. Zeinab Awad El-Sayed

  Department of Pediatrics, Ain Shams University (ASU)

International

- Prof. Angelo Ravelli

  Pediatrics, Department of Neurosciences, Rehabilitation, Ophthalmology, Genetics, and Maternal-Infantile Science (DiNOGMI), University of Geno, Italy.
- Prof. Lanny J. Rosenwasser

  Department of Medicine, University of Missouri-Kansas City School of Medicine, American Academy of Allergy, Asthma & Immunology (AAAI), USA, World Allergy Organization (WAO)

- Dr. Karen Brandt Onel

  Division of Pediatric Rheumatology, Hospital for Special Surgery (HSS), Clinical Pediatrics Weil Cornell Medicine, USA
- Prof. Robert F. Lemanske, Jr

  Institute for Clinical and Translational Research, University of Wisconsin School of Medicine and Public Health, USA
- Ass. Prof. Soad Hashad

  Pediatric Rheumatology, Tripoli University, Libya.
- Prof. Sami Bahna

  Pediatrics & Medicine, Allergy and Immunology Section, Louisiana State University Health Sciences Center, USA
- Prof. Seza Özen

  Department of Pediatric Rheumatology, Hacettepe University, Ankara, Turkey

Cardiology

- Prof. Alyaa A. Kotby

  Department of Pediatrics, Ain Shams University (ASU)

Critical Care/ Emergency Medicine

National

- Prof. Hatem Hussein

  Department of Pediatrics, Zagazig University (ZU)
- Prof. Khaled Talaat Muhammad

  Department of Pediatrics, Tanta University (TU)
- Prof. Mahmoud Taher Elmogy

  Department of Pediatrics, Al-Azhar University
- Prof. Mohammed Attia El-Bayoumi

  Department of Pediatrics, Mansoura University (MANS)
- Prof. Nabil Abdel-Aziz Mohsen

  Department of Pediatrics, Cairo University (CU)

- Prof. Tarek Abd El Gawwad

  Department of Pediatrics, Ain Shams University (ASU)

International

- Prof. Patrick Van de Voorde

  Department of Emergency Medicine, Ghent University Hospital, Belgium

  Belgian Society of Emergency & Disaster Medicine (BeSEDiM)

Endocrinology

National

- Prof. Basma Abd-Elmoez

  Department of Pediatrics, Minia University (MU)

- Prof. Eman Munir

  Department of Pediatrics, Ain Shams University (ASU)

- Prof. Hesham El Hefnawy

  National Institute of Diabetes and Endocrinology (NIDE).

- Prof. Magdy Omar

  Department of Pediatrics, Alexandria University (ALEXU)

- Prof. Nermine Salah

  Department of Pediatrics, Cairo University (CU)

International

- Prof. Joseph Wolfsdorf

  Department of Pediatrics, Harvard Medical School

  Boston Children's Hospital, USA

Gastroenterology

National

- Dr. Ahmed Megahed

  Department of Pediatrics, Mansoura University (MANS)

- Prof. Mohamed Attia Bayoumi

  Department of Pediatrics, Mansoura University (MANS)

- Dr. Mohamed El Guindy

Department of Pediatrics, Menoufia University

- Prof. Soheir Ibrahim

  Department of Pediatrics, Al-Azhar University

International

- Prof. Hania Szajewska

  Department of Pediatrics, The Medical University of Warsaw, Poland

- Prof. Hassan Hesham Hassan

  Department of Pediatrics, University of Arizona, Tucson, Arizona, USA

- Prof. Yvan Vandenplas

  KidZ Health Castle Children's Hospital, UZ Brussel University Hospital, Vrije Universiteit Brussel, Belgium

Hematology

- Prof. Ahmed Kaddah

  Department of Pediatrics, Cairo University (CU)

- Prof. Ahmed Mansour

  Department of Pediatrics, Mansoura University (MANS)

- Prof. Azza Abd El Gawad Tantawy

  Department of Pediatrics, Ain Shams University (ASU)

- Prof. Hoda Hassab

  Department of Pediatrics, Alexandria University (ALEXU)

- Prof. Iman Abdel Raouf

  Department of Pediatrics, Cairo University (CU)

- Prof. Khalil Abd El Khalek

  Department of Pediatrics, Cairo University (CU)

- Prof. Mervat Atfy

  Department of Pediatrics, Zagazig University (ZU)

- Prof. Mohamed Abdel-Mohsen

  Department of Pediatrics, Ain Shams University (ASU)

- Prof. Osama El Safy

Department of Pediatrics, Zagazig University (ZU)

- Prof. Seham Ragab

  Department of Pediatrics, Menoufia University

Neonatology

- Prof. Abdel Azim El Mazary

  Department of Pediatrics, Minia University (MU)
- Prof. Abd Ellatif Mohamed Abd Elmoez

  Department of Pediatrics, Assiut University (AUN)
- Prof. Mohamed Abdelmaaboud

  Department of Pediatrics, Minia University (MU)
- Prof. Mohamed Mohamed El Mazahi

  Department of Pediatrics, Al-Azhar University
- Prof. Nahed Fahmy Helal

  Department of Pediatrics, Cairo University (CU)
- Prof. Nehal Mohamed El Ragal

  Department of Pediatrics, Ain Shams University (ASU)
- Prof. Safaa Elmeneza

  Department of Pediatrics, Al-Azhar University

Nephrology

National

- Prof. Alaa Thabet

  Department of Pediatrics, Alexandria University (ALEXU)
- Prof. Amr Sarhan

  Department of Pediatrics, Mansoura University (MANS)
- Prof. Ihab El Hakim

  Department of Pediatrics, Ain Shams University (ASU)
- Prof. Moftah Mohamed Rabie

  Department of Pediatrics, Al-Azhar University
- Prof. Mohamad Eissa

Department of Pediatrics, Cairo University (CU)

- Prof. Neveen A Soliman

    Department of Pediatrics, Cairo University (CU)

- Prof. Sawsan Elmoselhy

    Department of Pediatrics, Ain Shams University (ASU)

International

- Dr. Federica Zotta

    University Department of Pediatrics, DPUO, Unit of Immune and Infectious Diseases, Bambino Gesù Children's Hospital and Research Institute (IRCCS), Rome, Italy.

- Prof. Pierre Cochat

    Centre de référence des maladies rénales rares Néphrogones, Hôpital Femme Mère Enfant, Hospices Civils de Lyon & Université de Lyon, Lyon, France, Former S.G. of IPNA

Neurology

- Prof. Azza Ahmed Eltayeb

    Department of Pediatrics, Assiut University (AUN)

- Prof. Bahaa El Sheikh

    Department of Pediatrics, Aswan University (ASWU)

- Prof. Hafez Mahmoud Bazaraa

    Department of Pediatrics, Cairo University (CU)

- Prof. Hanan Mohamed Ibrahim

    Department of Pediatrics, Ain Shams University (ASU)

Pulmonology

- Prof. Fatma ElHeneidy

    Department of Pediatrics, Cairo University (CU)

- Prof. Laila Abdel Ghaffar Hagazy

    Department of Pediatrics, Ain Shams University (ASU)

    Department of Pediatrics, Armed Forces College of Medicine, (AFCM)

- Prof. Mahmoud M. El-Zalabany

  Department of Pediatrics, Alexandria University (ALEXU)
- Prof. Magda Elseify

  Department of Pediatrics, Ain Shams University (ASU)
- Prof. Magdy Zidan

  Department of Pediatrics, Mansoura University (MANS)
- Prof. Nader Fasseh

  Department of Pediatrics, Alexandria University (ALEXU)
- Dr. Osama Taha Amer

  Department of Pediatrics, Zagazig University (ZU)
- Prof. Sherif Reda

  Department of Pediatrics, Al-Azhar University
- Prof. Tharwat Deraz

  Department of Pediatrics, Ain Shams University (ASU)

Additional Contributors *(other than Pediatrics)*

- Dr. Magdy El-Ekiaby, Consultant of Transfusion Medicine *(Member)*

  Blood Transfusion & Hemophilia Centre, Shabrawishi Hospital
- Prof. Mohamed Shehata

  Department of ENT, Ain Shams University (ASU)
- Prof. Salwa Youssef

  Department of Clinical Pathology, Hematology & Blood Transfusion,

  Ain Shams University (ASU)

  Egyptian Society of Transfusion Medicine

Methodology Reviewer

- Prof. Ivan D. Florez

  Department of Pediatrics, University of Antioquia, Medellin, Colombia

  School of Rehabilitation Science, McMaster University, Hamilton, Canada

  Leader of the AGREE Collaboration

  Director of Cochrane Colombia

EPG Group Photos

Group photographs of the early grand meetings of the EPG members and collaborators

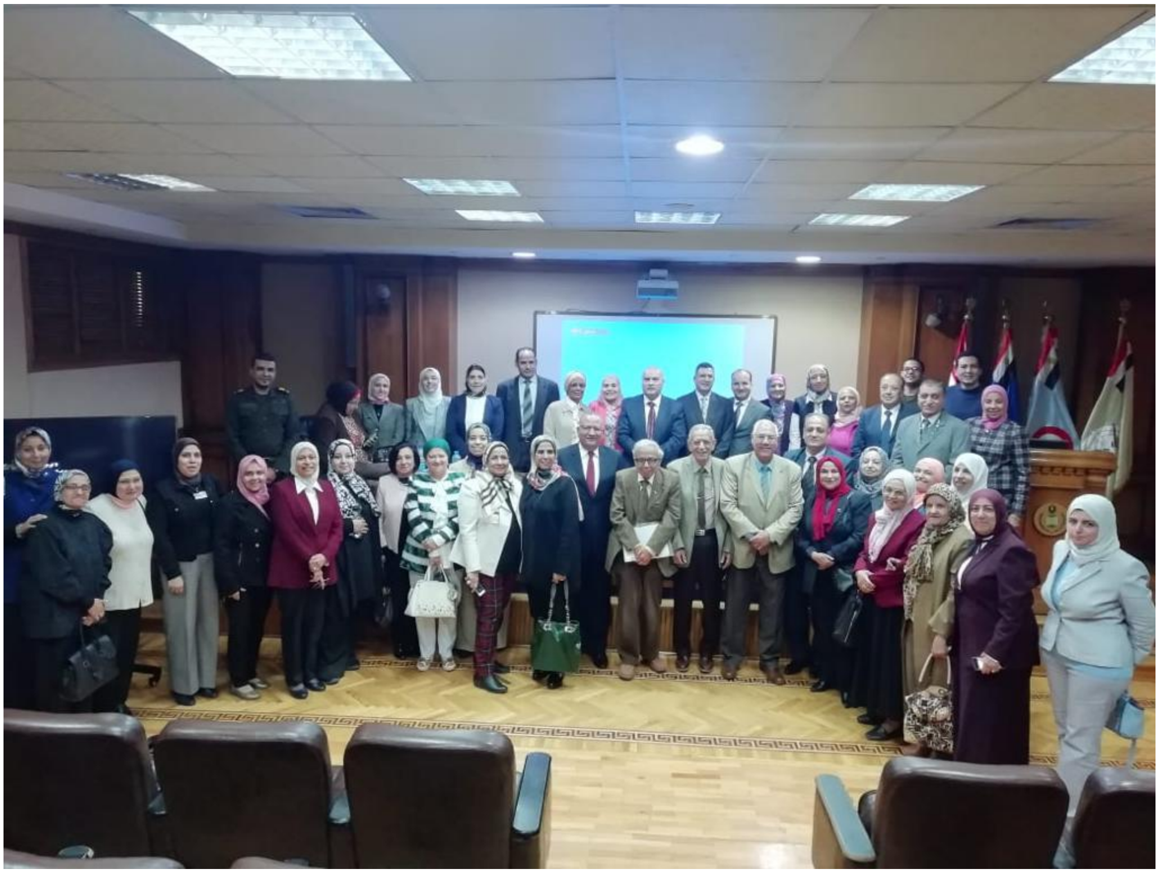

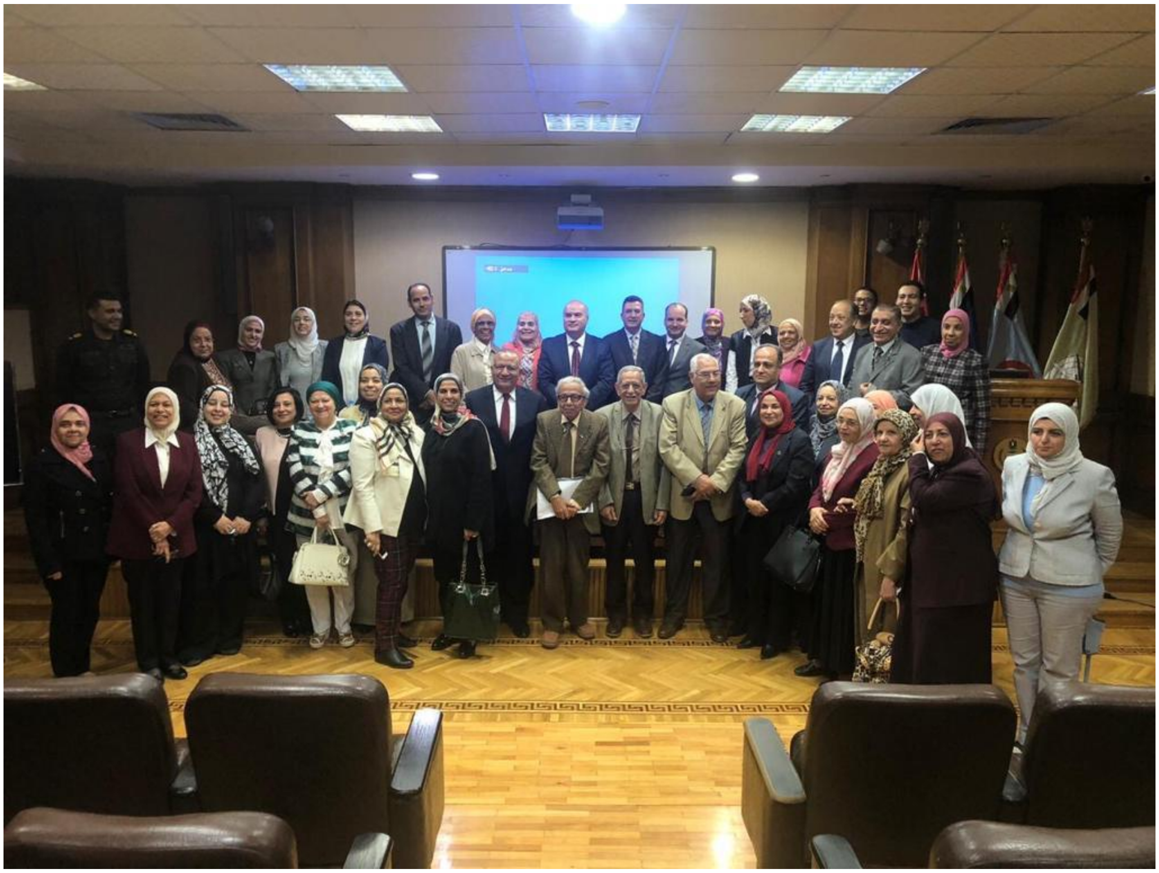

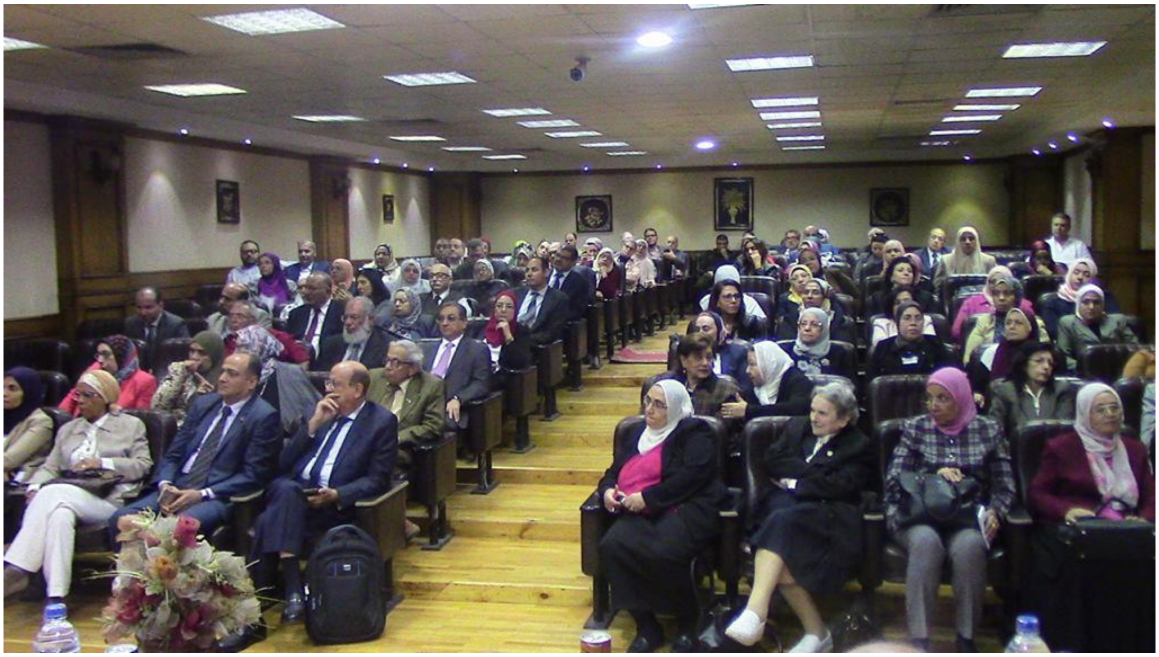

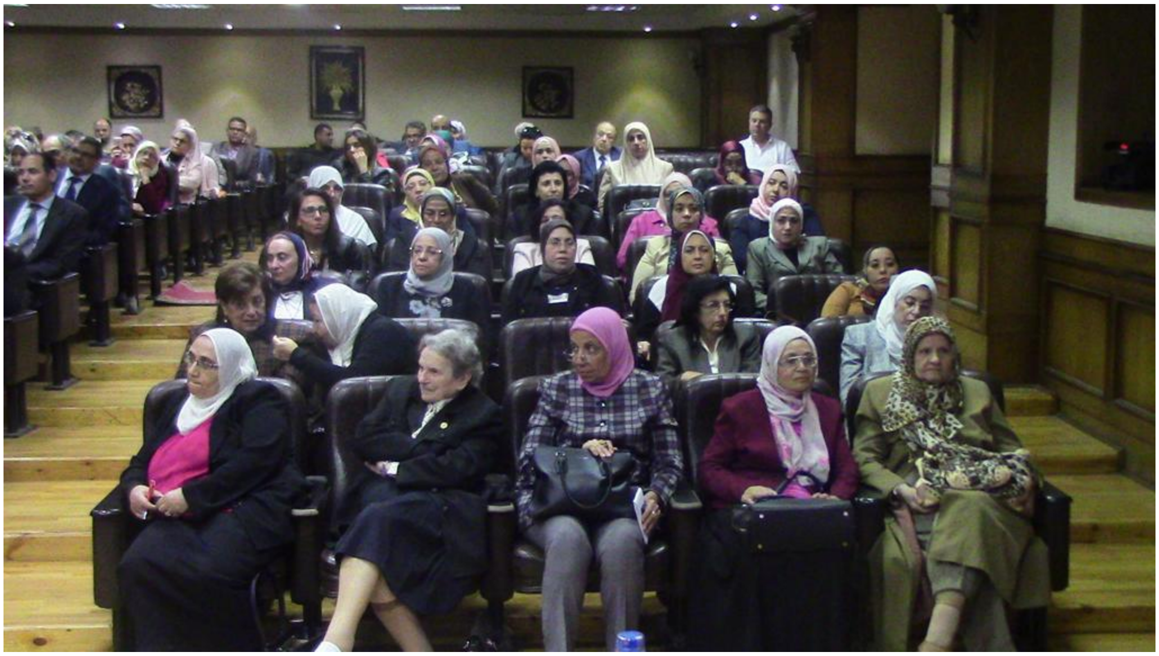

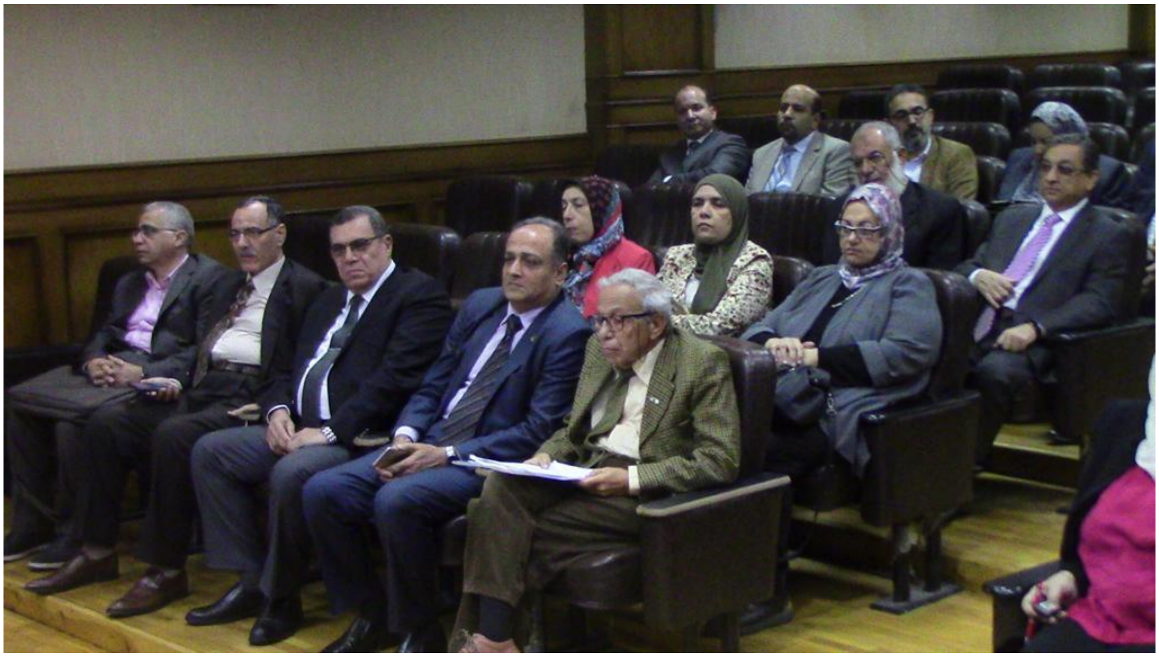

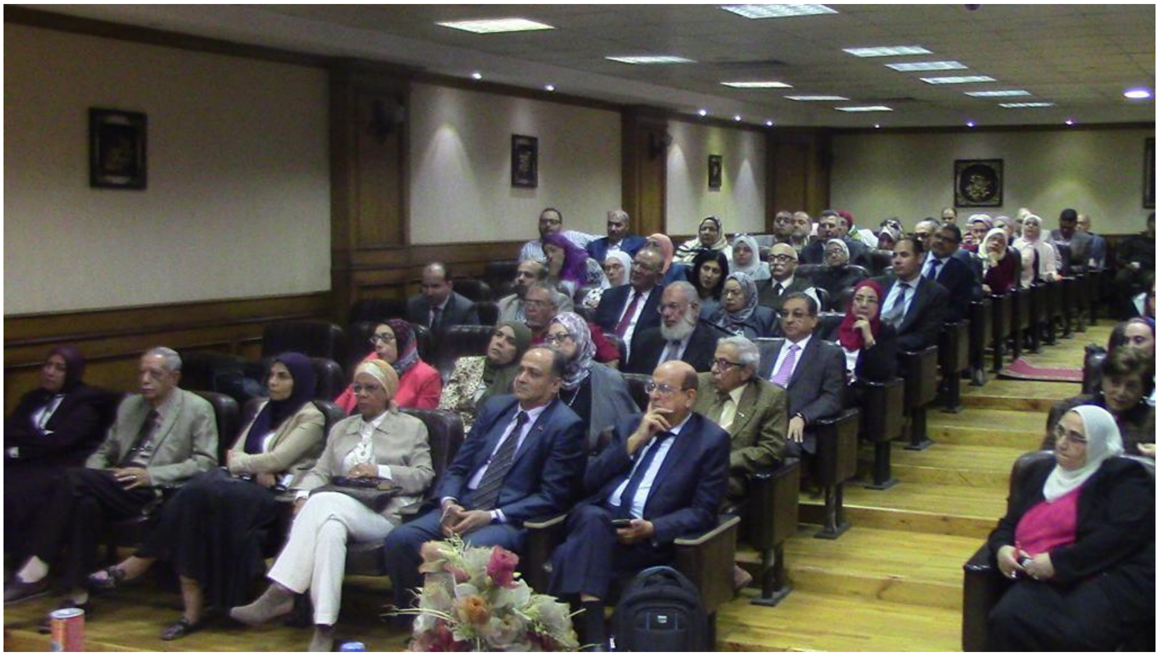

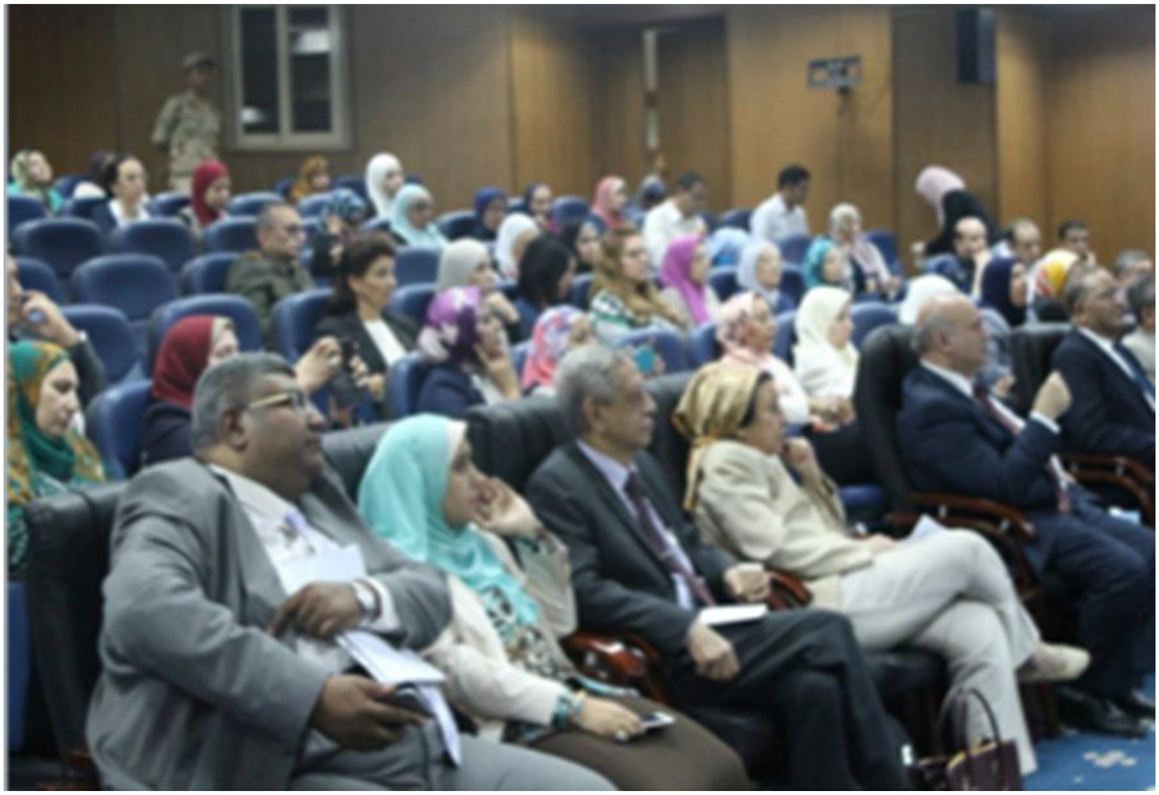

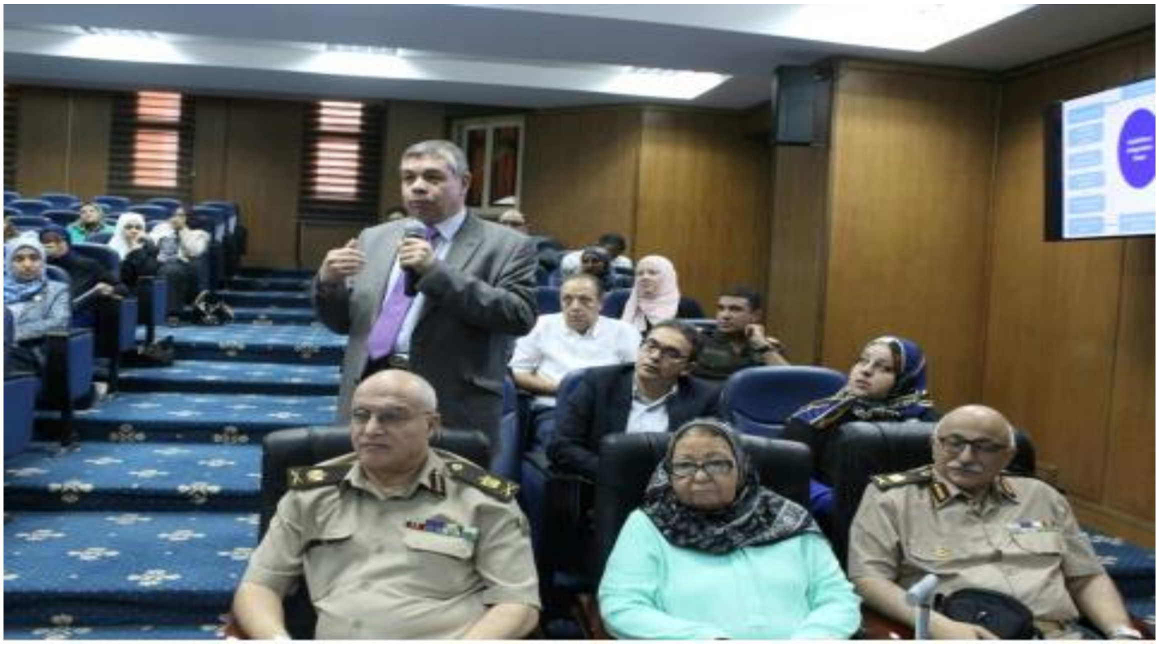

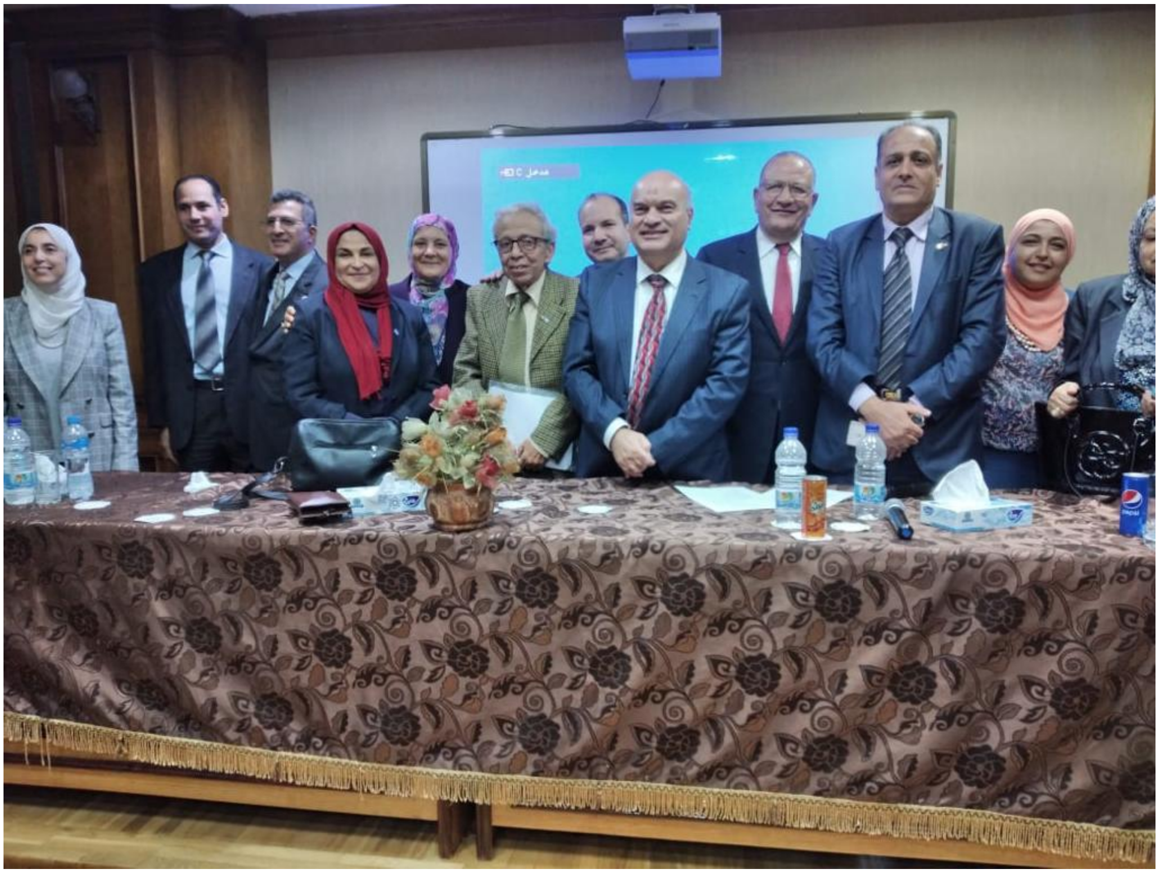

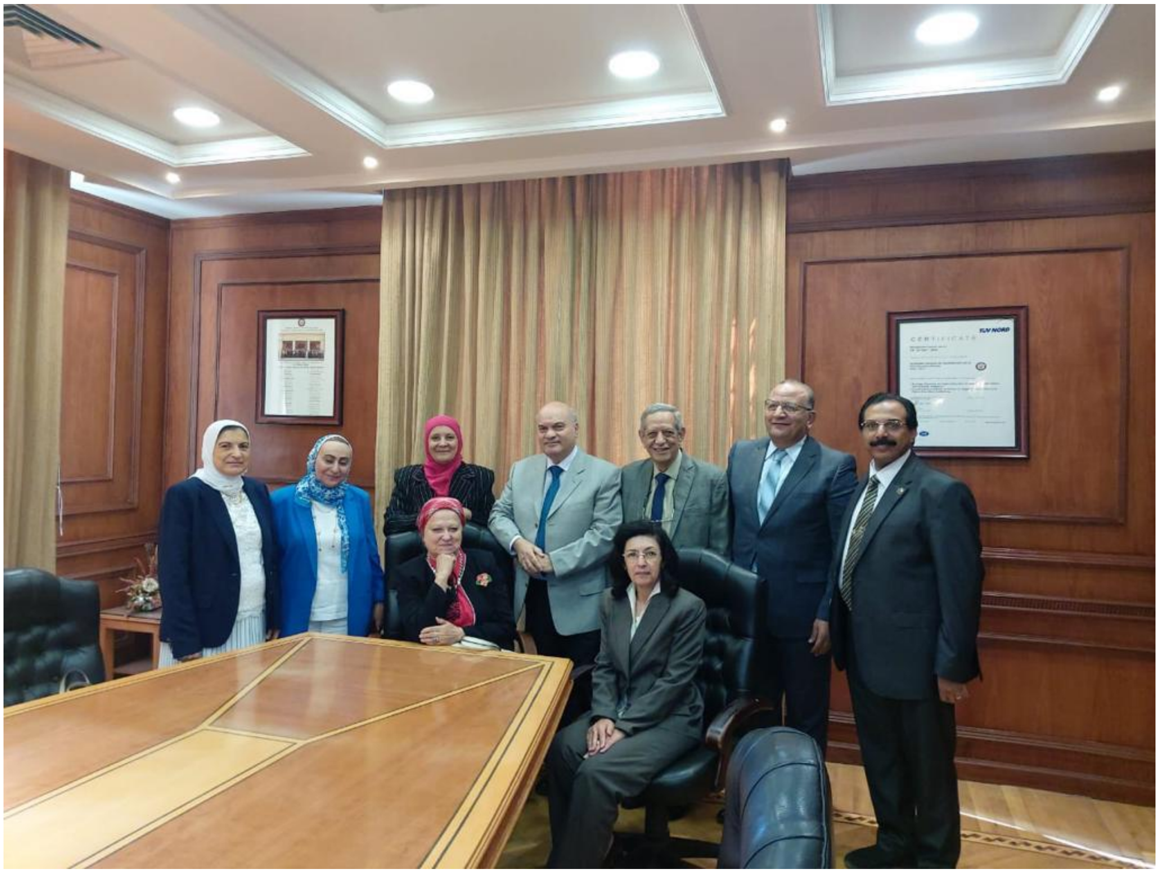

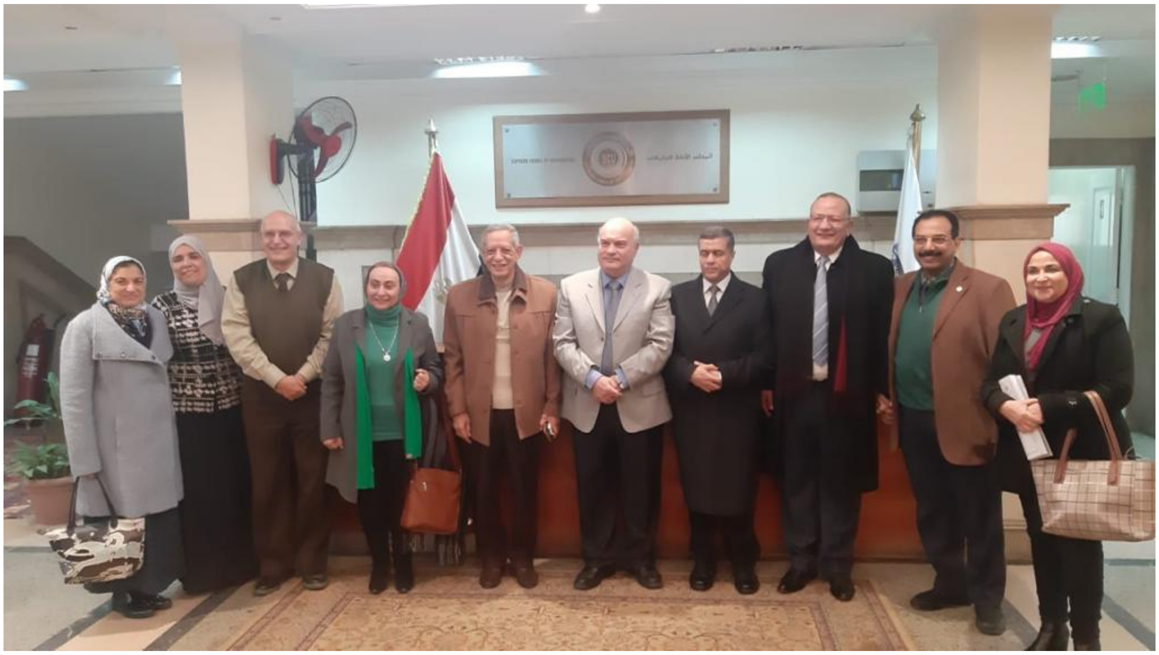

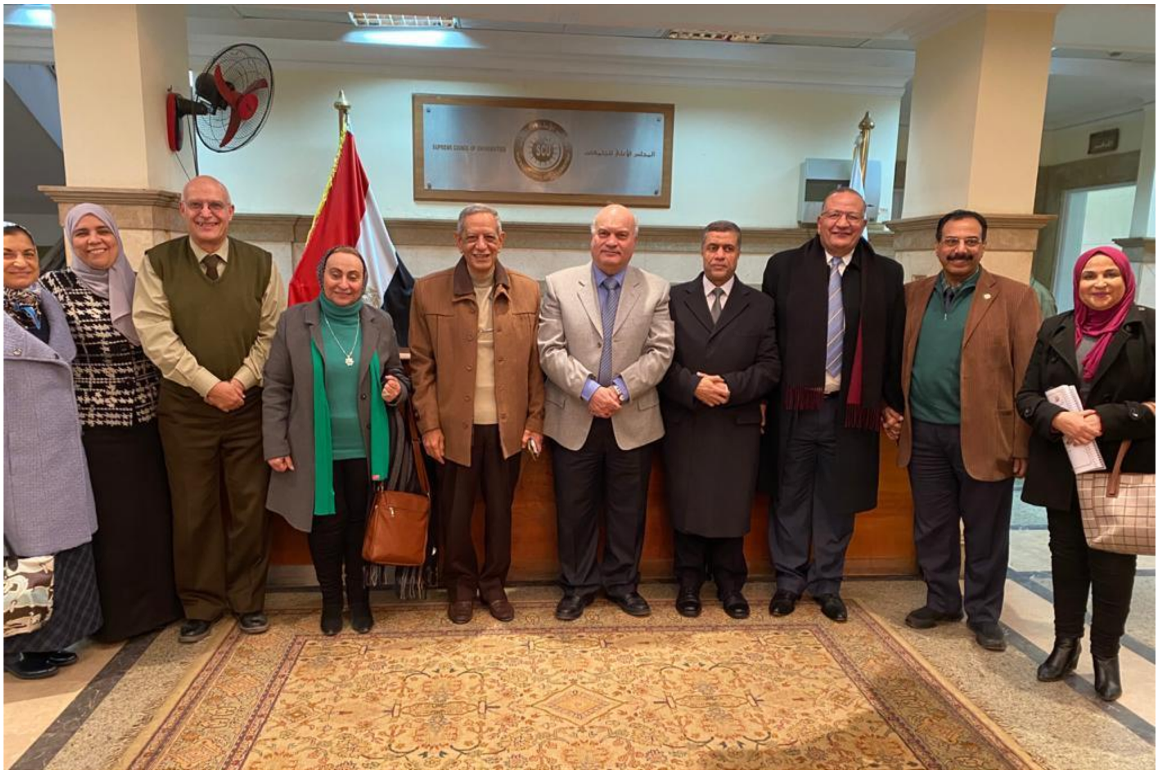

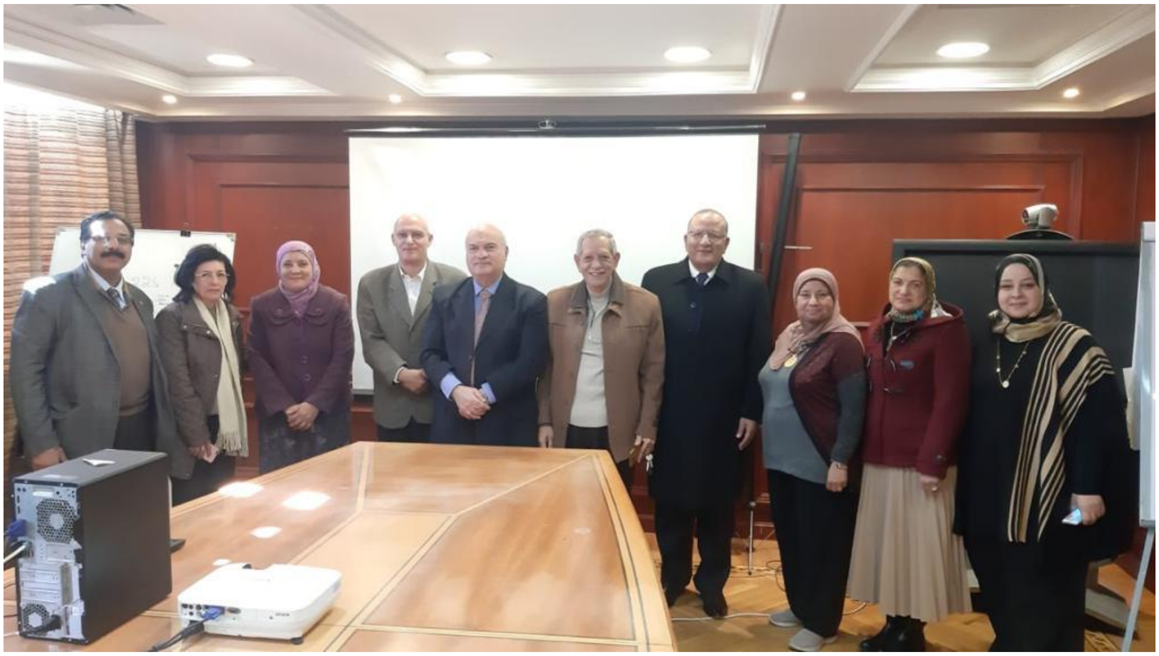

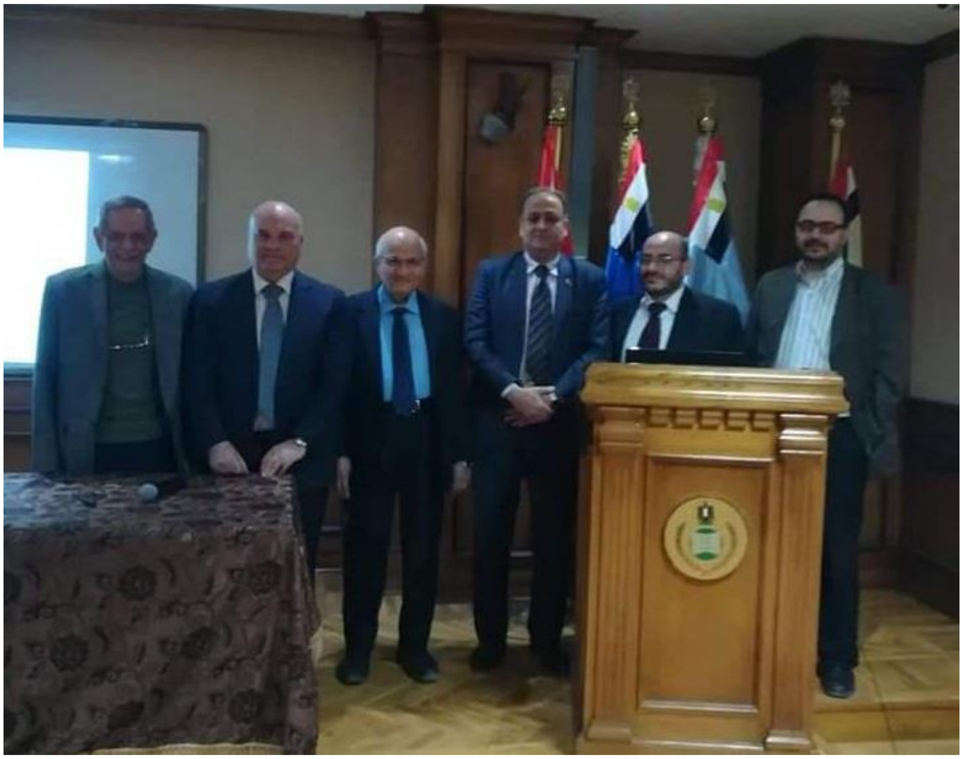

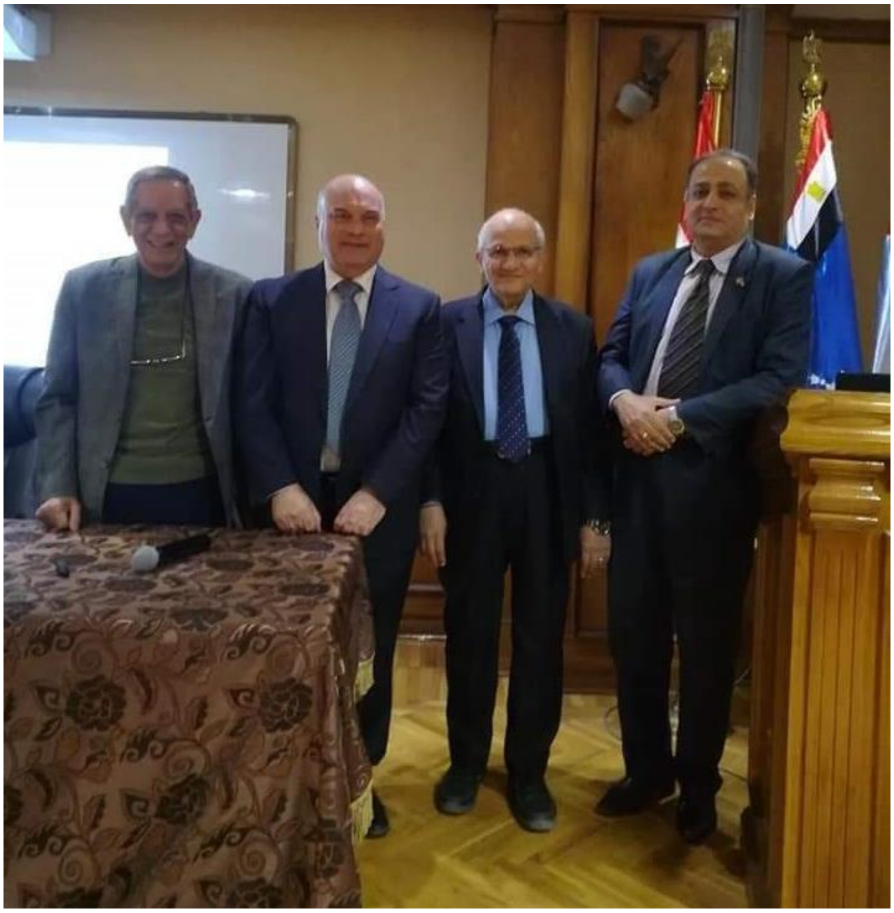

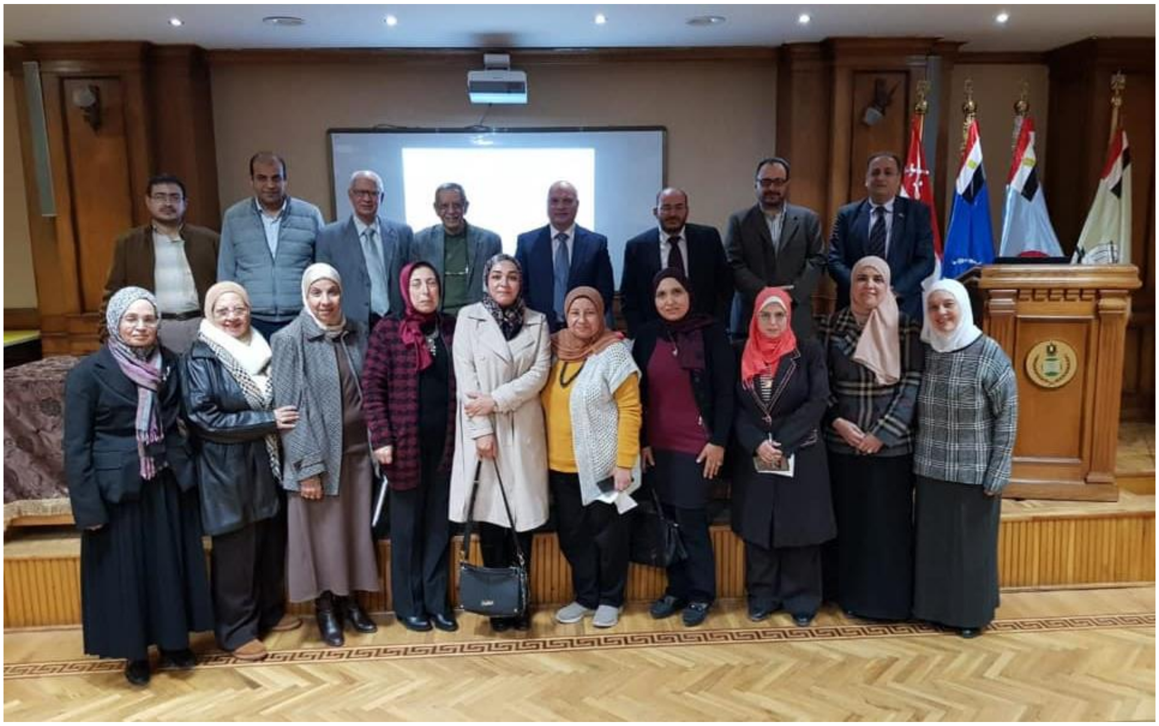

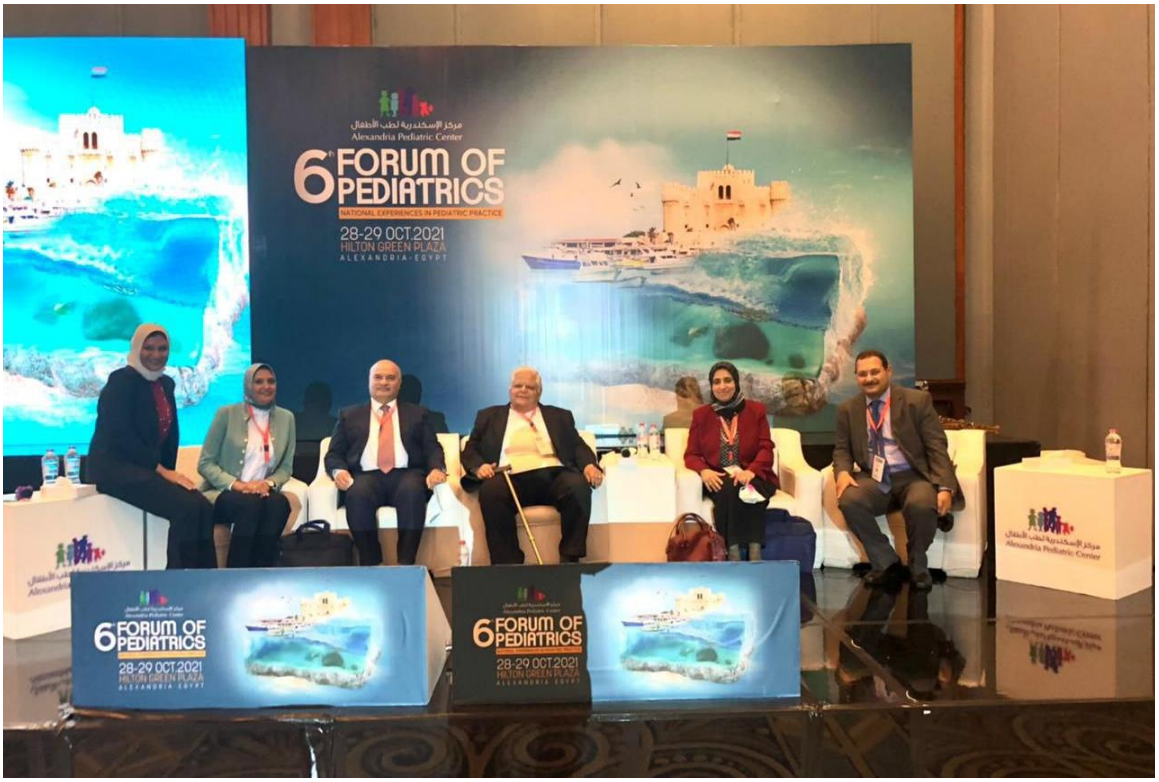
مركز الإسكندرية لطب الأطفال
Alexandria Pediatric Center
6th FORUM OF PEDIATRICS
NATIONAL EXPERIENCES IN PEDIATRIC PRACTICE
28-29 OCT.2021
HILTON GREEN PLAZA
ALEXANDRIA - EGYPT

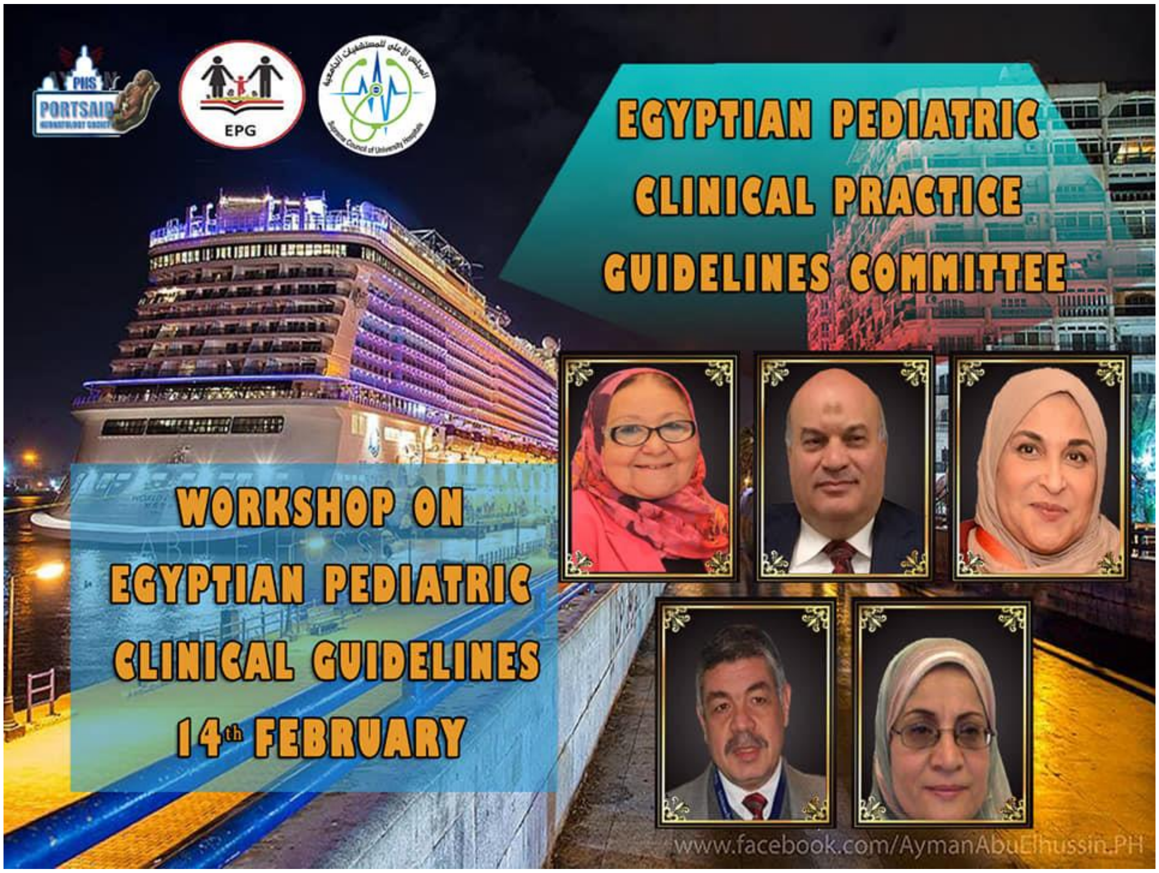
EPG
EGYPTIAN PEDIATRIC
CLINICAL PRACTICE
GUIDELINES COMMITTEE
WORKSHOP ON
EGYPTIAN PEDIATRIC
CLINICAL GUIDELINES
14th FEBRUARY
www.facebook.com/AymanAbuElhussin.PH

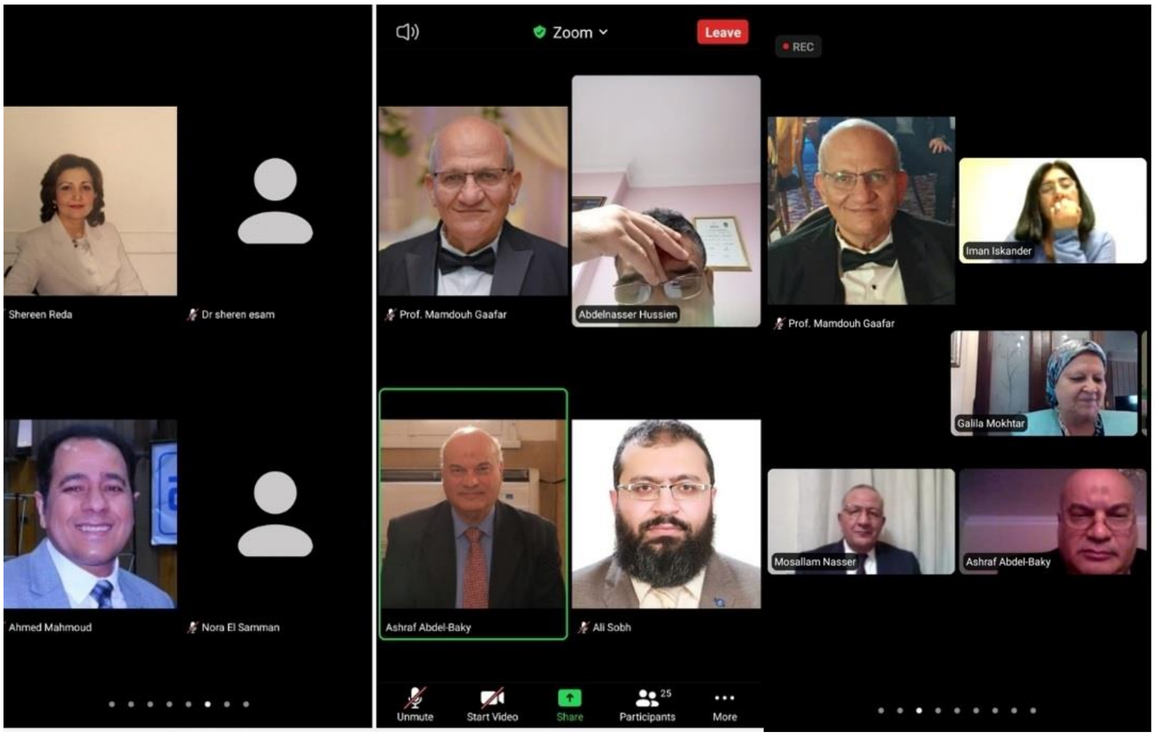
Shereen Reda
Dr sheren esam
Ahmed Mahmoud
Nora El Samman
Zoom
Leave
Prof. Mamdouh Gaafar
Abdelnasser Hussien
Ashraf Abdel-Baky
Ali Sobh
Unmute
Start Video
Share
25
Participants
More
REC
Prof. Mamdouh Gaafar
Iman Iskander
Galila Mokhtar
Mosallam Nasser
Ashraf Abdel-Baky

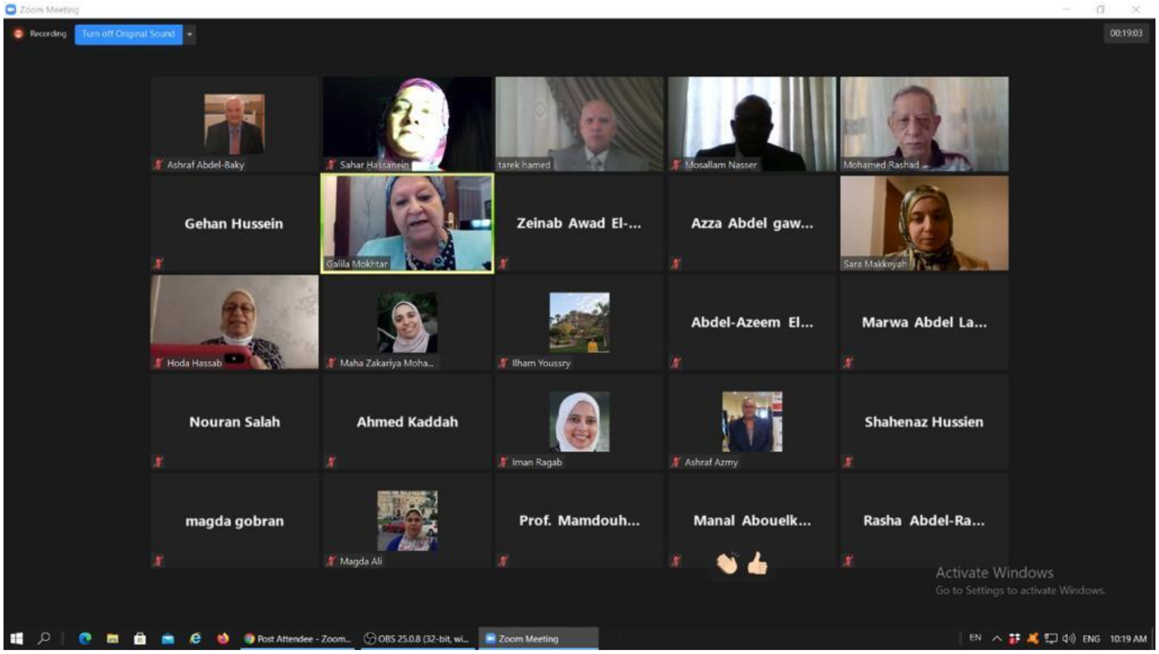
Zoom Meeting
Recording
Turn off Original Sound
00:19:03
Ashraf Abdel-Baky
Sahar Hassanein
tarek hamed
Mosallam Nasser
Mohamed Rashad
Gehan Hussein
Galila Mokhtar
Zeinab Awad El-...
Azza Abdel gaw...
Sara Makkeyah
Hoda Hassab
Maha Zakariya Moha...
Ilham Youssry
Abdel-Azeem El...
Marwa Abdel La...
Nouran Salah
Ahmed Kaddah
Iman Ragab
Ashraf Azmy
Shahenaz Hussien
magda gobran
Magda Ali
Prof. Mamdouh...
Manal Abouelk...
Rasha Abdel-Ra...
Activate Windows
Go to Settings to activate Windows.
Post Attendee - Zoom...
Zoom Meeting
ENG
10:19 AM

The participation of all Departments of Pediatrics in Egyptian Universities, the National Research Centre, and others were presented at the 2021 Online Guidelines International Network (GIN) Conference

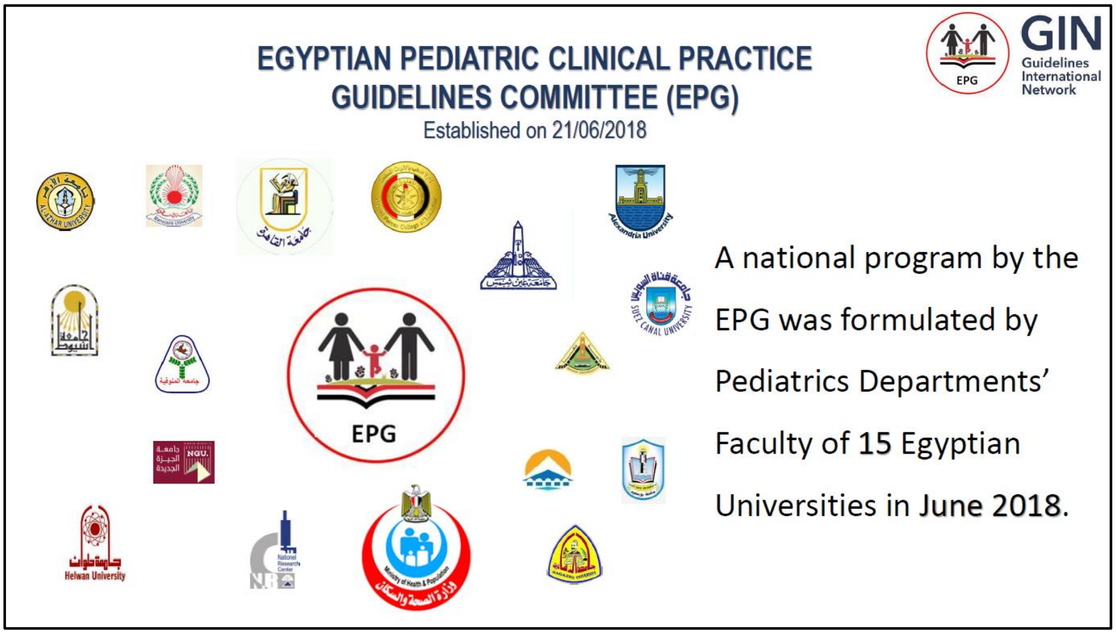

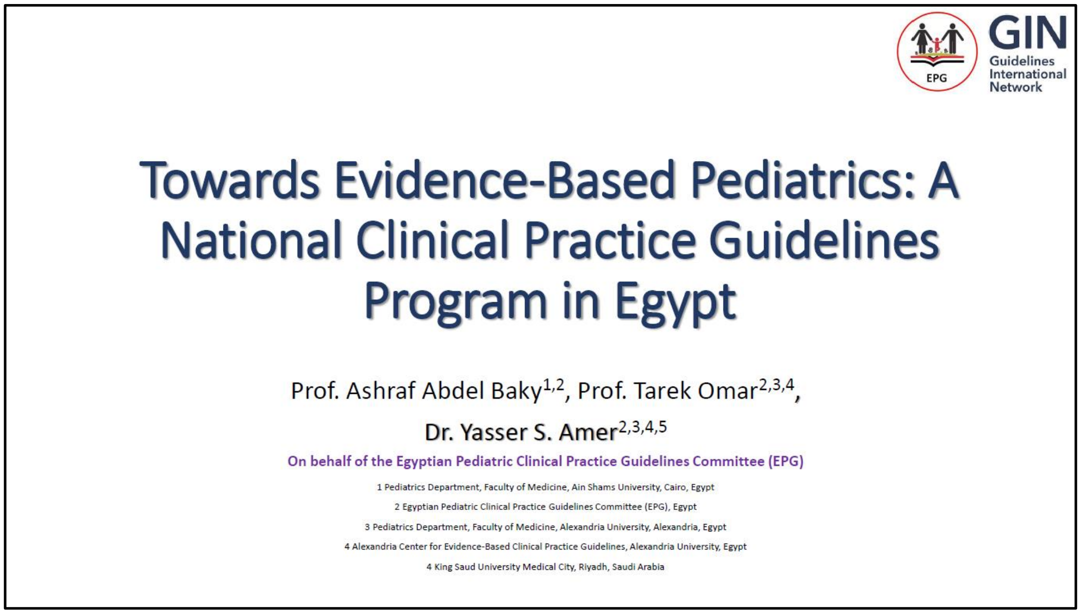

Supplement: Supplementary file 1 — Additional file 1. Recognition and Honor List. [file 42269_2023_1059_MOESM1_ESM.pdf]
